# Causes and consequences of end-Ediacaran extinction: An update

**DOI:** 10.1017/ext.2023.12

**Published:** 2023-05-15

**Authors:** Simon A.F. Darroch, Emily F. Smith, Lyle L. Nelson, Matthew Craffey, James D. Schiffbauer, Marc Laflamme

**Affiliations:** 1 Department of Earth and Environmental Sciences, Vanderbilt University, Nashville, TN, USA; 2 Evolutionary Studies Institute, Vanderbilt University, Nashville, TN, USA; 3 Senckenberg Research Institute and Museum of Natural History, Frankfurt, Germany; 4 Department of Earth and Planetary Sciences, Johns Hopkins University, Baltimore, MD, USA; 5 Department of Earth Sciences, Carleton University, Ottawa, ON, Canada; 6 Department of Biological Sciences, University of Nebraska, Lincoln, NE, USA; 7 Department of Geological Sciences, University of Missouri, Columbia, MO, USA; 8 X-Ray Microanalysis Laboratory, University of Missouri, Columbia, MO, USA; 9 Department of Chemical and Physical Sciences, University of Toronto Mississauga, Mississauga, ON, Canada

**Keywords:** Community ecology, ecosystem, evolution, macroevolution, volcanic eruptions

## Abstract

Since the 1980s, the existence of one or more extinction events in the late Ediacaran has been the subject of debate. Discussion surrounding these events has intensified in the last decade, in concert with efforts to understand drivers of global change over the Ediacaran–Cambrian transition and the appearance of the more modern-looking Phanerozoic biosphere. In this paper we review the history of thought and work surrounding late Ediacaran extinctions, with a particular focus on the last 5 years of paleontological, geochemical, and geochronological research. We consider the extent to which key questions have been answered, and pose new questions which will help to characterize drivers of environmental and biotic change. A key challenge for future work will be the calculation of extinction intensities that account for limited sampling, the duration of Ediacaran ‘assemblage’ zones, and the preponderance of taxa restricted to a single ‘assemblage’; without these data, the extent to which Ediacaran bioevents represent genuine mass extinctions comparable to the ‘Big 5’ extinctions of the Phanerozoic remains to be rigorously tested. Lastly, we propose a revised model for drivers of late Ediacaran extinction pulses that builds off recent data and growing consensus within the field. This model is speculative, but does frame testable hypotheses that can be targeted in the next decade of work.

## Impact statement

The majority of extinction-based paleontological research over the last four decades has focused on the ‘big 5’ mass extinctions of the Phanerozoic. In parallel, however, geologists and paleontologists working in the Precambrian have mulled the existence of one or more pulses of extinction (and potentially ‘mass extinction’) in the latest Neoproterozoic (~574–539 million years ago) shortly before the onset of the Cambrian. These episodes of global biotic turnover removed the mysterious Ediacara biota, as well as groups of more recognizable animal fossils. In this review, we summarize the history of ideas and research surrounding these events, as well as recent work in a range of fields that is attempting to identify the drivers – both biotic and abiotic – of extinction. We outline four key questions which, we argue, will help us to compare the causes and consequences of Ediacaran extinction alongside the Phanerozoic ‘Big 5’, and which will help us decide whether the ‘Big 5’ might eventually become the ‘Big 6’ (or the ‘Big 7’, if the current biodiversity crisis is considered). Finally, we propose a model for drivers of late Ediacaran extinction that builds off recent data. This model is speculative, but frames testable hypotheses that will help determine the role these events may have played in the Ediacaran–Cambrian emergence of the modern-looking biosphere, and thus the extent to which Ediacaran extinction and the Cambrian explosion may be linked.

## Introduction

The Ediacaran–Cambrian (E–C) transition arguably marks the most important geobiological revolution of the past billion years, characterized by large perturbations to global geochemical cycles, a permanent step-change in the character of the sedimentary record, the rise of macroscopic eukaryotic life, and potentially one or more pulses of mass extinction. Although all aspects of this interval have been the subjects of intense research efforts over the last three decades, the existence of putative biotic turnover events in the latest Ediacaran has received particular attention. These events may not only have played a crucial role in fueling evolutionary radiation during the ‘rise of animals’ and acted as a powerful influence on the appearance of metazoan ecosystems with a more modern-looking structure (Knoll and Carroll, 1999; Droser et al., 2017; Darroch et al., 2018a,b), but they also have invited comparison with the ‘Big 5’ mass extinctions of the Phanerozoic (Raup and Sepkoski, 1982), and thus may yield more general lessons about the causes and consequences of these catastrophic events in Earth’s history.

Five years ago, Darroch et al., 2018a, summarized the evidence for one or more pulses of extinction in the late Ediacaran and presented a series of key questions that would be crucial for driving knowledge forward in this field. Since then many of these questions have been explored, with new paleontological, geochemical, and geochronological datasets providing the scaffolding required for building our understanding of this interval. In this review we summarize recent work surrounding the end-Ediacaran extinction events, examine the extent to which the questions posed in 2018 have been answered, and propose new questions, challenges, and research avenues that will continue to illuminate the changes that occurred over the E–C transition.

## Ediacaran fossils and early animals

**Figure 1.**
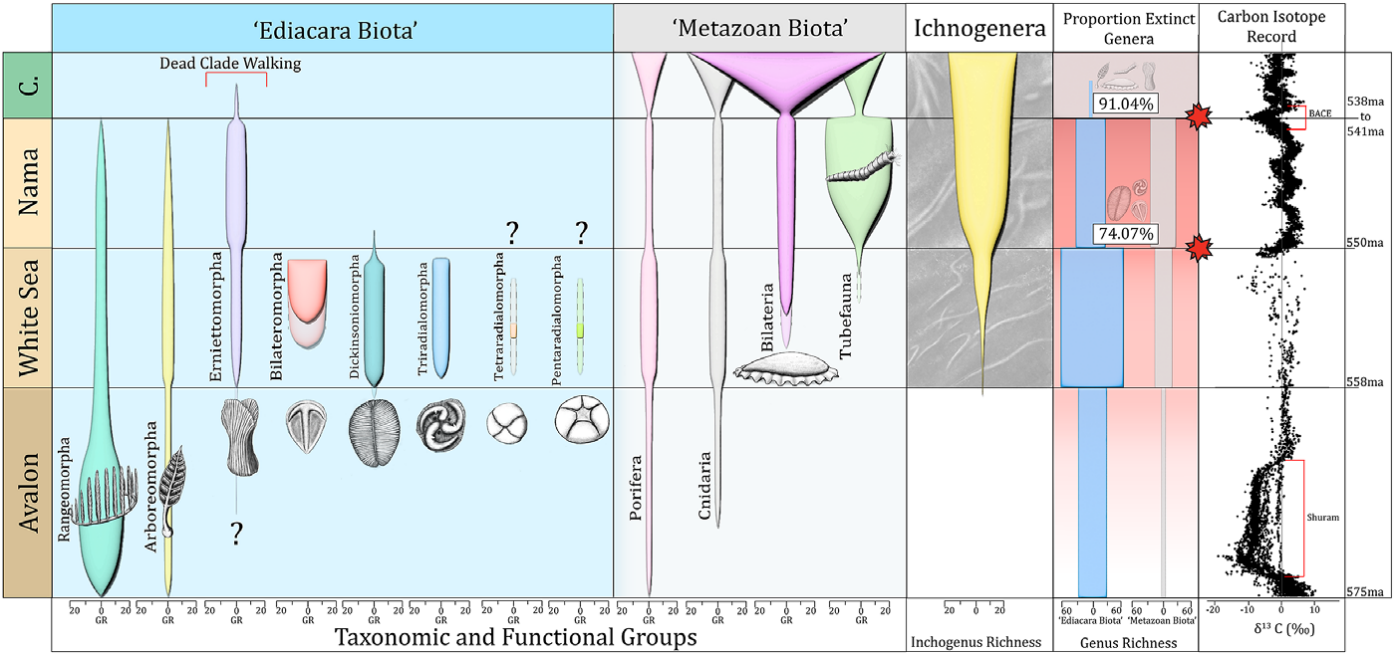
Updated summary figure illustrating the stratigraphic distribution and diversity among groups of Ediacara biota, as well as metazoans, bilaterian ichnogenera, and a δ^13^C curve (compiled from Yang et al. (2021), Bowyer et al. (2022), and references therein). The stratigraphic ranges of the Pentaradialomorpha and Tetraradialomorpha are currently uncertain, but currently constrained by a detrital zircon age of 556 ± 24 Ma obtained from the Bonney Sandstone in South Australia (Ireland et al., 1998). Solid colors represent minimum age estimates (where available), while shaded regions represent uncertain range estimates where taxa are found beneath (or between) dated horizons. Extinction intensities – as percentage of genera lost – are given for the two putative extinction pulses at the White Sea-Nama and the E–C boundaries; intensities were calculated by simply measuring the proportion of surviving genera over total genera in the preceding assemblage zone (although see discussion in the text surrounding problems with calculating these transition).

The late Ediacaran is characterized by the presence of macroscopic body fossils that are typically categorized as belonging to one of two faunas: either (1) ‘Ediacara biota’ – an enigmatic collection of soft-bodied organisms with uncertain relationships to extant animal phyla, and which have been subdivided into morphogroups (see, e.g., Erwin et al., 2011; Laflamme et al., 2013); or (2) true metazoans – referring to fossils that can be more readily allied with living animal groups. However, this subdivision within Ediacaran organisms is becoming increasingly more obsolete with recent developmental (Gold et al., 2015), and phylogenetic (Dunn et al., 2021) data suggesting that many representatives of the Ediacara biota are likely stem-group members of known eumetazoan clades. On the other hand, given that these two different categories of organisms appear and disappear at different times in the fossil record (see, e.g., Figure 1) and possess strong morphological differences (including, for example, the presence/absence of a body plan that is present among extant phyla), classifying Ediacaran-aged taxa as ‘Ediacara biota’ vs. ‘metazoans’ is arguably still useful, and provides a heuristic model with which to explore their faunal dynamics. So, while we refer to ‘Ediacara biota’ and metazoans over the course of this review, we emphasize that this does not preclude members of the Ediacara biota as belonging to animal clades.

## History

The history of thought surrounding the existence of end-Ediacaran extinction events is closely linked to work defining, dating, and characterizing the base of the Cambrian. Early attempts to define this boundary were spearheaded by decades of dedicated stratigraphic, paleontological, and geochronological studies conducted by the International Geoscience Programme (IGCP), reviewed nearly 30 years ago by Brasier et al. (1994). Nevertheless, these efforts were overwhelmingly focused on the subsequent ‘explosion’ of animal phyla in the early Cambrian, rather than the disappearance of Ediacaran soft-bodied organisms below the boundary. This is perhaps not surprising given that, prior to propositions by Seilacher (1984, 1985, 1989, 1992), the Ediacara biota were overwhelmingly interpreted as belonging to extant metazoan groups (e.g., Glaessner, 1984; Gehling, 1991). As such, the fossil record of the E–C transition could be satisfactorily explained as the result of taphonomic biases towards the preservation of biomineral shells, teeth, and bones (Gehling, 1999). In contrast, Seilacher (1989) was largely alone in arguing that many, if not all, Ediacaran fossils represented neither true metazoans nor their earliest stem ancestors, and therefore must have suffered an extinction at some point during the E–C transition.

The case for extinction was reinvigorated by chemostratigraphic studies that identified multiple, large carbon isotope fluctuations in the late Neoproterozoic potentially tied to major upheavals in the carbon cycle; one that particularly stands-out as potentially coeval with extinction is a negative excursion that reaches values as low as −9 ‰, known now as the basal Cambrian carbon isotope excursion (or ‘BACE’), which coincides with the E–C boundary (Kirschvink et al., 1991; Knoll and Walter, 1992; Narbonne et al., 1994). In the decade that followed the “Decision on the Precambrian-Cambrian boundary stratotype” (Brasier et al., 1994), notable discussion on a transitional Ediacaran–Cambrian extinction arose. For instance, noting parallels with the Permo-Triassic boundary, Knoll and Carroll (1999) stated a clear case for a mass extinction separating the Ediacaran and Cambrian faunas – a case that was only strengthened with the recognition that the BACE event also coincided with the global and synchronous disappearance of biomineralizing fossils that characterize the latest Ediacaran (Amthor et al., 2003). The presence of earlier extinction events, however, only became recognized with more focused biostratigraphic work, and, in particular, attempts to stratigraphically subdivide the late Ediacaran.

Waggoner (1999, 2003) identified three broad communities of Ediacara biota, which are still broadly thought to represent three chronologically and environmentally distinct assemblages. From oldest to youngest these are: (1) the Avalon Assemblage (~574–558 Ma), characterized by deep-water communities (Narbonne, 2005; Liu et al., 2015); (2) the White Sea Assemblage (~558–550 Ma), which represents the apex of diversity and disparity among Ediacara biota (Grazhdankin, 2004; Droser and Gehling, 2015); and (3) the Nama Assemblage (~550–538 Ma), which records a drop in the diversity of Ediacara biota, alongside an expansion in several modes of metazoan ‘ecosystem engineering’ including increased trace fossil diversity, the advent of macroscopic biomineralization, and widespread suspension feeding (Germs, 1972; Wood and Curtis, 2014; Schiffbauer et al., 2016; Darroch et al., 2018a,b). Due to the apparent loss in diversity among Ediacara biota, the transition from the White Sea assemblage to the Nama assemblage has also been suggested as recording an extinction event. Although discussion surrounding biotic turnover at the White Sea–Nama transition has intensified recently (e.g., Darroch et al., 2018a,b; Tarhan et al., 2018; Muscente et al., 2019; Evans et al., 2022), the formerly recognized ‘Kotlinian crisis’ in the southern Urals and East European Platform, which removed diversity among Ediacara biota prior to the appearance of biomineralizing metazoans like *Cloudina* (Brasier, 1992), may be time-equivalent with this transition (Grazhdankin, 2014).

The last 10 years have seen an abundance of work on putative late Ediacaran extinction events, focusing on the possible causes and consequences of extinction pulses, as well as to what extent extinction may be linked to the Cambrian explosion. Key work in this area is summarized below.

## Late Ediacaran bioevents and extinction models

**Figure 2.**
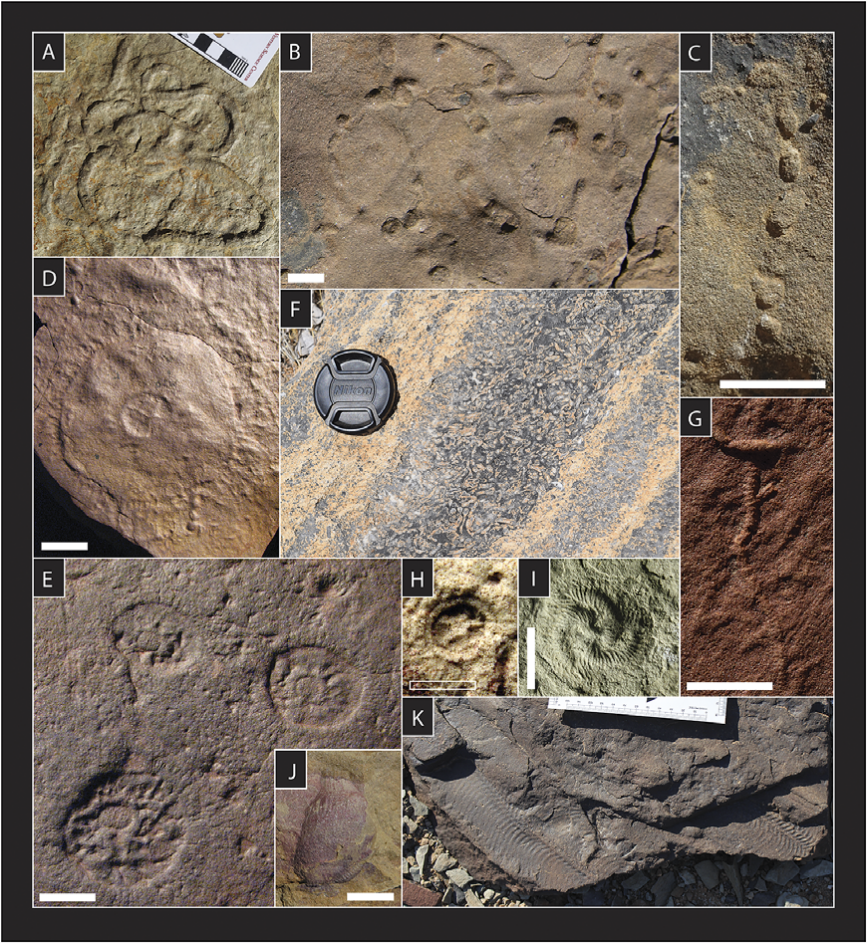
Putative late Ediacaran ecosystem engineers, including bilaterian tracemaking behaviors that involve sediment ‘bulldozing’ and biomixing (A - *Parapsammichnites*), bioirrigation (B-C - large treptichnids), and suspension feeders such as *Paleophragmodictya* (D-E; sp. nos. P32338 and P32332-P32352 respectively, South Australia Museum), biomineralizing *Cloudina* (F), and other unidentified tubefauna (G). Lastly, many Ediacara biota may have also had important ecosystem engineering impacts; the enigmatic taxa *Ernietta* (J), *Arkarua* (H; sp. no. P26768, South Australia Museum), *Tribrachidium* (I; sp. no. N3993/5056, Palaeontological Institute, Moscow) and *Pteridinium* (K) are all also thought to have functioned as suspension feeders, and thus played a crucial role in forging energetic links between the pelagic and benthic realms (Cracknell et al., 2021; Darroch et al., 2022). Specimens shown in A–C, F, G, J and K from the Nama Group of southern Namibia (all Urusis Fm., with the exception of *Ernietta* shown in J from the Dabis Fm.), and photographed in the field. Filled scale bars = 1 cm, open scale bars = 5 mm.

Darroch et al. (2018a) argued for two pulses of Ediacaran extinction: one between the White Sea and Nama assemblages at ~550 Ma, and another at the E–C boundary itself. This inference was supported by Muscente et al. (2019), who used a network analysis of fossil communities together with their associated paleoenvironments to demonstrate that turnover was unlikely to be the result of a secular facies bias (see also Evans et al., 2022). The first extinction pulse apparently removed many of the most charismatic (and enigmatic) groups of Ediacara biota best known from White Sea-aged fossil localities in South Australia and Russia – principally the dickinsoniomorphs, triradialomorphs, tetraradialomorphs, and bilateromorphs – leaving a relatively species-poor assemblage dominated by erniettomorphs, arboreomorphs, and rangeomorphs in Nama-aged strata. Interestingly, genus richness among White Sea and Nama-aged rangeomorphs is also substantially lower than from the Avalon, suggesting that, while the rangeomorphs survived the first pulse of extinction, they were nonetheless negatively impacted. In contrast, genus richness among the erniettomorphs is equivalent or even potentially higher in the Nama, suggesting a positive response to the removal of White Sea-aged shallow-marine biocoenoses, and/or to the environmental conditions that pervaded the terminal Ediacaran. Although extinction is markedly focused within specific groups of Ediacara biota, many metazoan genera present in the White Sea were also affected (Figure 1). In concert with the disappearance of Ediacara biota, the first extinction pulse is also marked by the widespread appearance of more recognizable metazoans, including increased diversity (and/or behavioral disparity) in bilaterian tracemakers (Mángano and Buatois, 2014; Darroch et al., 2021), the appearance of tube-dwelling animals with debated affinities (see, e.g., Schiffbauer et al., 2020; Shore et al., 2020), calcifying and sessile lophotrochozoans (Shore et al., 2021), and rare body fossils of segmented bilaterians plausibly representing early annelids or panarthropods (Chen et al., 2019) (Figure 2). Unlike representatives of the Ediacara biota, these organisms can be more confidently allied with groups and lineages that persisted into the Cambrian (e.g., Yang et al., 2016, 2020). Given the apparent vermiform character of much of this Nama-aged metazoan fauna, whether preserved as body- or trace fossils, Schiffbauer et al. (2016) referred to this interval as ‘Wormworld’.

An apparent second extinction pulse occurs at the E–C boundary, demarcated by the disappearance of almost all remaining Ediacara biota, as well as much of the metazoan fauna that characterizes the Nama Assemblage (in particular the calcifying taxa *Cloudina* and *Namacalathus* (Amthor et al., 2003; Smith et al., 2016, as well as cosmopolitan tube-dwelling forms such as *Shaanxilithes* and *Gaojiashania* (Zhu et al., 2017)). We note that estimates of extinction intensity – particularly with respect to tube-dwelling taxa – over this pulse is complicated by a lack of consensus in taxonomic studies, and thus to what extent latest Ediacaran and earliest Cambrian tubefauna may be related. For example, recent studies suggest that some Ediacaran-type biomineralizing taxa may persist into the early Cambrian (e.g., Zhu et al., 2017; Yang et al., 2021), albeit in limited localities and numbers (Cai et al., 2019). By way of contrast, few late Ediacaran taxa have unambiguously been identified from the Cambrian, and many that do are tentative descendants (e.g., cambroctoconids), and/or re-appear in considerably modified form (see, e.g., Park et al., 2021). In general, more focused systematic work on the affinities of, and relationships between, late Ediacaran and early Cambrian tubefauna is sorely needed (Schiffbauer et al., 2022).

In terms of what may have driven these two events, Darroch et al., 2018a, discussed evidence for two hypotheses – termed ‘catastrophe’ and ‘biotic replacement’ – representing the summation of ideas and data given in previous studies (principally Amthor et al., 2003; Erwin and Tweedt, 2012; Laflamme et al., 2013; and Darroch et al., 2015). The ‘catastrophe’ model suggested that Ediacaran extinction events were driven by environmental perturbations, reflected in the negative carbon isotope excursions during the E–C transition. This model invokes parallels with several of the Phanerozoic ‘Big 5’ extinctions, in particular those coinciding with the Permian–Triassic and Triassic-Jurassic boundaries. In contrast, ‘biotic replacement’ suggested that the extinction events were instead driven by the emergence of new metazoan ecosystem engineering behaviors and their associated downstream geobiological impacts, which permanently altered marine environments in a fashion that was deleterious to soft-bodied Ediacara biota. There are fewer clear parallels for this process in the Phanerozoic; however, one hypothesized cause of the late Devonian mass extinction centers on the initial radiation of terrestrial forests, which significantly influenced weathering patterns leading to eutrophication, anoxia, and prolonged intervals of ecological stress (Algeo and Scheckler, 1998; Lu et al., 2021). This model, therefore, identifies the emergence of new ecosystem engineers as the ultimate driver of mass extinction, albeit through a complex series of terrestrial–marine teleconnections (e.g., Lu et al., 2021). Other studies have focused on the impact of humans as ecosystem engineers and as a driver of the on-going ‘6th mass extinction’ (e.g., Yeakel et al., 2020; Pineda-Munoz et al., 2021), potentially raising interesting parallels between the first and most recent mass extinctions of macroscopic life. In general, the extent to which the emergence of new ecosystem engineering behaviors in deep time had led to extinction, or instead evolutionary radiations, is a question that has long required more focused work (see, e.g., Erwin, 2008).

## Do these intervals of biotic turnover represent (‘mass’) extinctions at all?

Our understanding of the geochronology, chemostratigraphy, and biostratigraphy of the late Ediacaran has increased substantially in the last 10 years. However, given uncertainties surrounding stratigraphic correlation between sites (Xiao et al., 2016), the placement of key boundaries (Nelson et al., 2022), and the mechanisms of fossil preservation (Laflamme et al., 2013; Slagter et al., 2022; Gibson et al., 2023), a reasonable question is: do these apparent intervals of biotic turnover really represent extinction intervals (or more specifically, ‘mass’ extinctions) at all? The iconic Ediacaran fossil sites in South Australia illustrate some of these issues; they are among the best-studied Ediacaran localities, are frequently taken to epitomize the diversity and community structure of ‘White Sea’-aged assemblages, and almost always make their way into analyses of Ediacaran diversity through time (see, e.g., Laflamme et al., 2013; Darroch et al., 2015; Darroch et al., 2018b; Eden et al., 2022; Evans et al., 2022). However, beyond post-dating the Shuram-Wonoka isotope excursion and a U**–**Pb detrital zircon age of 556 ± 24 Ma obtained from the underlying Bonney Sandstone (Ireland et al., 1998), the age of the principal fossiliferous horizons in the Ediacara Member are unconstrained. Given that South Australia preserves a number of Ediacaran morphogroups thought to be disappeared over the White Sea-Nama transition, this is perhaps not a trivial barrier to inferring an extinction event.

Mass extinctions are typically identified as episodes of anomalously high rates of taxonomic loss, occurring on global scales, that are approximately synchronous over a relatively short interval of geological time (exactly how ‘short’ is an evolving field, but currently thought to be ~10^5^ years; see Burgess et al., 2014). We argue that the White Sea-Nama transition and E–C boundary currently satisfy one of these three criteria – specifically, being a global vs. regional signal. Older work (Laflamme et al., 2013; Boag et al., 2016) has suggested – and more recent work (Boddy et al., 2022; Evans et al., 2022) has confirmed – that there are no obvious geographical, facies, or preservational biases that can readily explain the loss of taxa, nor account for the observation that entire morphogroups (dickinsonimorpha, bilateromorpha, etc.) are lost, and thus extinction is apparently taxonomically clustered. The number and wide geographic spread of dated Nama-aged fossil sites also provides some support for dismissing the notion that White Sea-aged communities persist and remain widespread into the Nama. With respect to the issue with South Australian fossils mentioned above, it is thus far more likely that these communities are White Sea in age (coeval with well-dated horizons in Russia), rather than a totally unique Nama-aged locality.

The question as to rates (and magnitude) of taxonomic loss is harder to address. Calculating the simple proportion of surviving genera (over total genera in the preceding assemblage zone) gives genus extinction intensities of 74.1% for the White Sea-Nama (WS–NM) transition, and 91% over the Nama-Fortunian (NM–FN) – magnitudes noted by previous studies as being comparable to those estimated for many of the ‘Big 5’ (see, e.g., Evans et al., 2022). However, raw percentages are strongly biased by variations in sampling intensity, and quantifying extinction rates over the late Ediacaran is fraught with other difficulties stemming from the character of the Ediacaran fossil record, and ongoing difficulties with stratigraphic correlation and subdivision. For example, from our occurrence dataset (Figure 1; taxa and references provided in Supplementary Material) we can argue for major extinctions across the White Sea-Nama and E–C boundaries utilizing a simple proportion of extinct genera, but we do not have much confidence in Ediacaran per capita extinction rates using common methods such as Foote (1999), Alroy (2008), or Alroy (2014). The principal issue is that these methods utilize the proportion of taxa crossing boundaries for their extinction rate estimates in various patterns (e.g., boundary crossers, 3-timers, and gap fillers spanning 3 intervals with no detections in the middle) and the Ediacaran is dominated by taxa that occur in a single assemblage zone. This likely results from limited sampling, high turnover, and the long duration of these zones (~10–15 Ma). Thus, the effective sample size for an analysis of per capita Extinction rates across the Ediacaran is very small, leading to estimates of per capita extinction at the E–C boundary that have dubious reliability (for example: 2.48 per Foote, 1999, 3.04 per Alroy, 2014, utilizing the R package ‘divDyn’ Kocsis et al., 2019). The most direct quantitative solution would be to examine extinction rates at finer timescales than assemblage zones, but there is – as yet – no unifying framework for subdividing these intervals, and there is limited chronostratigraphic data to applying such a framework across global collections. We can only argue, for now, for the presence of high extinction at the E–C transition based on apparent turnover patterns, but providing firm quantitative support will require additional work, and (potentially) the application of other methods for extinction rate modeling.

The last criterion – that extinction is rapid and synchronous – is, similarly hard to satisfy, and something discussed in more detail below (see the section titled ‘Key questions in 2023’).

Another recent challenge to the existence of late Ediacaran extinction events has come from phylogenetic modeling, framing biotic patterns over the E–C transition as an artifact of evolutionary patterns and stem- vs. crown-group diversity dynamics (i.e., suggesting that Ediacaran extinction and Cambrian explosion are different facets of the same process). For example, Budd and Mann (2020) have used birth-death models to argue that, if all Ediacara biota are viewed as stem-group members of extant bilaterian clades (and assuming a high level of background extinction), then the proportion of diversity within the total group can be quickly ‘drowned’ by the crown, thus mimicking a mass extinction. However, these models invoke a consistent rate of background extinction, and so cannot explain the synchronous and global loss of multiple morphogroups over, for example, the White Sea–Nama transition. Consequently, these models do not provide a good match for the observed diversity trends, although we note that Budd and Mann’s (2020) models incorporating mass extinction events do provide a match, and so may be relevant for strikingly different reasons.

## Key questions in 2018

Darroch et al. (2018a) emphasized that the ‘catastrophe’ and ‘biotic replacement’ models were not mutually exclusive, but noted that each bring contrasting predictions, and moreover could be tested by addressing four key questions. Below, we briefly re-cap these questions (along with appropriate context), before reviewing recent work in these areas and assessing to what extent these questions have been answered.

*1. What, and when, was the Shuram?* In the Phanerozoic, several of the ‘Big 5’ mass extinctions are associated with perturbations to global geochemical cycles, which in turn are recorded in global isotope records (see, in particular, large igneous provinces as drivers of the Permian–Triassic (Shen et al., 2011) and Triassic–Jurassic (Ruhl et al., 2011) mass extinctions). Consequently, the existence of a large carbon isotope excursion in the late Neoproterozoic -– the Shuram event – has long been suspected as a potential source of environmental stress (see discussion in Tarhan et al., 2018). The Shuram is among the largest negative carbon isotope excursions in Earth history, with carbonate δ^13^C values as low as −12‰ recorded on multiple paleocontinents (e.g., Grotzinger et al., 2011). It has been proposed that this excursion records massive perturbations to global geochemical cycles (e.g., Fike et al., 2006), which could plausibly represent a source of physiological stress and a possible driving mechanism for late Ediacaran extinction. However, in 2018 there were poor radiometric age constraints for the onset, duration, and recovery from the Shuram excursion, limiting efforts to test for temporal correlation with extinction. Furthermore, there was disagreement over whether the excursion should be interpreted as a primary marine (e.g., Husson et al., 2015) or diagenetic (e.g., Knauth and Kennedy, 2009) signal.

*2. What was the BACE?* Similar to the Shuram, the BACE is a large negative carbon isotope excursion (δ^13^C values < −6‰) recorded in multiple localities worldwide. In 2018 the BACE had better age constraints than the Shuram and was recognized as coinciding with a second extinction pulse at the E–C boundary (Amthor et al., 2003). Like the Shuram, however, there were outstanding questions as to what the excursion represented, what its precise timing and duration was, and whether it was primary or diagenetic, locally or globally controlled. All of these uncertainties limited interpretations for causal linkages between excursion and extinction.

*3. Can we disentangle correlation* versus *causation in late Ediacaran extinction events?* This question builds from the previous two in emphasizing the need for plausible cause and effect in extinction studies – a standard set by workers over the last decade on the Permian–Triassic extinction involving integrated geochronology, geochemistry, and paleontology (e.g., Shen et al., 2011; Burgess et al., 2014; Clarkson et al., 2015). In 2018, although there was potential temporal correlation when discussing both the ‘catastrophe’ and ‘biotic replacement’ models, evidence for causation was lacking. For example, with the ‘catastrophe’ model there was little idea as to what E–C carbon isotope excursions represented, precluding discussion of links between environmental change and sources of biotic stress. Likewise, with ‘biotic replacement’ there were significant knowledge gaps surrounding how the Ediacara biota and emerging metazoan fauna interacted, both as individual taxa and within communities. Consequently, there was minimal evidence for biotic interactions – antagonistic or otherwise – and thus no substantiated mechanism for a biotic driver of extinction.

*4. What role did the end-Ediacaran extinction play in the Cambrian Explosion?* Several of the Phanerozoic ‘Big 5’ mass extinctions were followed by radiation in surviving clades, expansions of morphologic disparity, and rapid diversification of new taxa (e.g., post-K/Pg radiations of mammals (O’Leary et al., 2013), birds (Ksepka et al., 2017), and mollusks (Krug and Jablonski, 2012). An intriguing question, therefore, is to what extent the Cambrian explosion could have been triggered (or perhaps driven) by late Ediacaran extinction pulses as a response to an ‘ecological vacuum’ (e.g., Knoll and Carroll, 1999).

## Have these questions been answered?

Questions 1 and 2 (‘what, and when, were the Shuram and BACE isotope excursions?’) have arguably received the most attention over the last five years, with new geochemical and geochronological data bringing these events into sharper focus. With respect to the Shuram, recent Re-Os dates from Northwest Canada and Oman have demonstrated that: (1) on separate paleocontinents, the excursions are synchronous within the error of these radioisotopic measurements, and (2) the excursion lasted <6.7 ± 5.6 million years from c. 574 Ma to c. 567 Ma (Rooney et al., 2020). Additional radioisotopic and chemostratigraphic data from Newfoundland are consistent with this finding and suggest that the Shuram carbon isotope excursion began after 571 Ma and ended before 562 Ma (Canfield et al., 2020). These new data demonstrate that this perturbation did not coincide with the White Sea–Nama transition (at c. 550 Ma (Bowring et al., 2007), as previously suggested, and, furthermore, demonstrate that it postdated both the Gaskiers glaciation and the earliest dated macrofossils of the Avalon assemblage (Macdonald et al., 2013; Pu et al., 2016; Matthews et al., 2021).

While the ultimate cause of the Shuram excursion remains contentious, many have suggested that mechanisms implicate changes in marine redox conditions (e.g., Fike et al., 2006; Zhang et al., 2019; Li et al., 2020) and/or changes in the locus of primary productivity (e.g., Busch et al., 2022). These changes are not mutually exclusive and could relate to external factors such as fluctuations in eustatic sea level (Busch et al., 2022) and/or nutrient availability (Cañadas et al., 2022). Other work has challenged the interpretation that large Neoproterozoic carbon isotope excursions record the global dissolved inorganic reservoir composition, but suggest they are still coeval responses to external forcings, such as primary production and sea level changes (e.g., Ahm et al., 2019). Regardless of its origin, at present it seems unlikely that the cause of the Shuram played any role in driving late Ediacaran extinction pulses (save, perhaps, for a decline in acanthomorphic acritarch assemblages (Ouyang et al., 2019; Yang et al., 2021), but may – interestingly – have played a role in driving origination.

**Figure 3.**
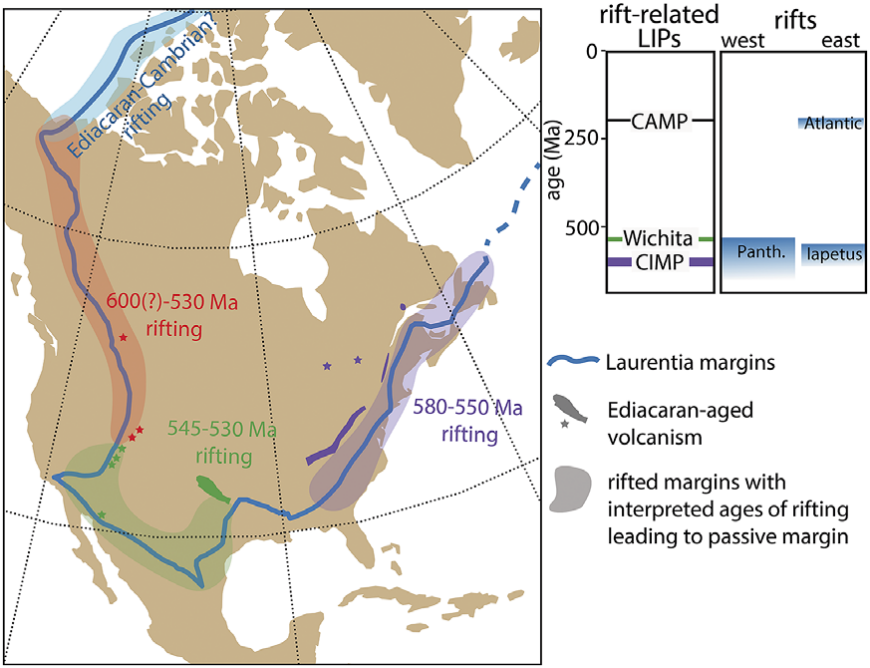
Ediacaran–Cambrian rift volcanism in Laurentia and timing of interpreted rifts leading to passive margin development around Laurentia after the breakup of Rodinia. Rifting along the southwestern margin has recently been suggested to coincide with the E–C boundary, potentially resulting in the BACE negative carbon isotope excursion and a second pulse of Ediacaran extinction (see Hodgin et al., 2021; Smith et al., 2022). CAMP, Central Atlantic magmatic province; CIMP, Central Iapetus magmatic province; Panth, Panthalassa Ocean.

While a link between the Shuram excursion and first pulse of extinction at the White Sea–Nama transition is irreconcilable with new radioisotopic constraints, Yang et al. (2021) have provided evidence for a second negative excursion that postdates the Shuram and ended at ~550 Ma. This excursion is potentially correlative with the carbon isotope excursion documented in the basal Nama Group of southern Namibia >548 Ma (Bowring et al., 2007; Wood et al., 2015) and/or negative carbon isotope values documented in the Stirling Quartzite of Death Valley, California (Verdel et al., 2011). The existence of a large negative excursion coincident with the White Sea-Nama transition would invite suggestions of causality, although more data will be required to establish beyond doubt the existence of two large negative carbon isotope excursions in the middle Ediacaran. Furthermore, Eden et al. (2022) found no evidence for a ‘catastrophe’-type signature in Nama-aged communities – something that might support a causal relationship with extinction. Nevertheless, the rift-related Central Iapetus magmatic province (CIMP)—with loosely constrained pulses from c. 580–550 associated with the opening of the Iapetus Ocean (Youbi et al., 2020)—provides a prospective mechanism for environmental and/or carbon isotope perturbations in the middle Ediacaran, worthy of further investigation (Figure 3).

With respect to the BACE, although large, negative carbon isotope excursions have been found at horizons approximately coinciding with the E–C boundary on several paleocontinents, there remain significant challenges with correlations and temporal calibration (e.g., Bowyer et al., 2022). Beyond understanding the carbon isotope record, these correlations have significant bearing on how the biostratigraphic records of the E–C boundary interval are interpreted, and thus our understanding of rates and patterns of biotic turnover at this boundary remains limited. Previous calibration of the BACE (and of the E–C boundary) at ~541 Ma hinged on a U–Pb ID-TIMS ash bed date just below onset of the negative carbon isotope excursion in the Ara Group of Oman (Bowring et al., 2007). However, more recent data from Namibia, South Africa, and northern Mexico have revised this calibration (Linnemann et al., 2019; Hodgin et al., 2021; Nelson et al., 2022). Nelson et al. (2022) suggest that, given available age constraints, the BACE and the last appearance of Ediacaran fossils, such as cloudinomorphs and erniettomorphs should be interpreted as <538 Ma. Regardless of its absolute age, as postulated when it was first identified in the 1990s, the BACE continues to hold up as a useful marker of the E–C boundary (e.g., Narbonne et al., 1994; Corsetti and Hagadorn, 2000). Furthermore, more recent data continue to reinforce that the BACE postdated most occurrences of Ediacaran fossils such as erniettomorphs and cloudinomorphs (e.g., Smith et al., 2022) and predated most occurrences of Cambrian trace fossils and small shelly fossils (e.g., Topper et al., 2022), supporting hypotheses of biotic turnover across this excursion.

These issues with correlation feed into discussion of what may have caused the BACE and to what extent it represents a plausible driver for extinction. For example, if emerging geochronological data reinforce the existing difficulties with correlating the BACE across different continents, then this might suggest that it represents a regional or diagenetic signal. We note, however, that the excursion persists across regional sequence boundaries and dolomitization fronts and is thus inconsistent with a purely diagenetic origin (Smith et al., 2022). Assuming that the excursion represents a perturbation to the global marine carbon cycle, Hodgin et al. (2021) suggested a genetic relationship with rift-related volcanism in southern Laurentia. This hypothesis suggests that dissolved inorganic carbon of marine waters attained highly ^13^C-depleted compositions due to the combined influx of mantle carbon from volcanic outgassing and combusted organic carbon within intruded sedimentary rift-basins, drawing parallels between end-Ediacaran extinction and the Permian–Triassic and Triassic-Jurassic mass extinction events. While this would be consistent with a ‘catastrophe’ model for the end-Ediacaran extinction, the proposed mechanistic link remains speculative, as it hinges on temporal correlation between the BACE and pulses of volcanism associated with the c. 539.5–530.0 Ma Wichita igneous province (Hodgin et al., 2021; Wall et al., 2021), as well as stratigraphic correlation to other basaltic and volcaniclastic units in southwestern North America (e.g., Smith et al., 2022; see Figure 3).

This work addressing questions 1 and 2 has naturally fed into question 3 – disentangling correlation vs. causation – in tying the late Ediacaran carbon isotope excursions to potential sources of ecological stress, and thus targeting the ‘catastrophe’ model for late Ediacaran extinction. However, substantial work has also been done in this vein targeting the ‘biotic replacement’ model, which suggests that the disappearance of the Ediacara biota was a consequence of ecosystem engineering by the emerging metazoan fauna. Two key questions in this regard have therefore been: (1) how intense was ecosystem engineering in the Ediacaran? And (2) how did the Ediacara biota and metazoan fauna interact?

In terms of the former, much recent effort has focused on the trace fossil record. Not only do bioturbating animals have powerful effects on resource flows and in modifying the physical environment (Rhoads et al., 1978; Jones et al., 1994; Rosenberg et al., 2001; Meysman et al., 2006), but they are also a record of ecosystem engineering that is relatively easy to preserve as fossils (Marenco and Bottjer, 2007). A detailed search through Nama-aged sediments in several localities worldwide has uncovered a remarkable diversity of Ediacaran ichnotaxa (e.g., Parry et al., 2017; Buatois et al., 2018; Turk et al., 2022), while other studies have shown that the impacts of these behaviors potentially reach Cambrian levels millions of years before the E–C boundary (e.g., Cribb et al., 2019). When set alongside the apparent low genus diversity of Nama-aged Ediacara biota (Laflamme et al., 2013; Darroch et al., 2015; 2018a,b), this would seem to lend strong support for the ‘biotic replacement’ model – at least over the White Sea-Nama transition (i.e., the first extinction pulse). However, there remains uncertainty surrounding the extent to which these ecosystem engineering impacts affected soft-bodied Ediacara biota, and thus to what extent there is a predictable pattern of extinction selectivity (Darroch et al., 2021).

This point dovetails with the latter, centered on ecological interactions between the Ediacara biota and metazoan fauna. The growing recognition that bed-penetrative bioturbation is much more pervasive and extends further back into the Ediacaran than previously thought (e.g., Jensen et al., 2000; Mángano and Buatois, 2020; Nelson et al., 2022; Turk et al., 2022), could suggest that the removal of microbial matgrounds might have been a plausible source of ecological stress and reduction of taphonomically favorable depositional settings. However, not only is there little evidence that matgrounds disappeared over the course of the E–C transition (Buatois et al., 2014), but in addition, frondose Ediacara biota – potentially the group(s) most reliant on matgrounds – are among the taxa that persist right up until the base of the Cambrian. Other hypotheses surrounding the ecosystem engineering impacts of bioturbation also seem to be inconsistent with patterns of extinction and survival over the White Sea-Nama transition, although are hampered by an incomplete understanding of how many groups of Ediacara biota functioned (Darroch et al., 2021).

Finally, some work has been done in the area of Question 4 (‘What role did the end-Ediacaran extinction play in the Cambrian Explosion?’), although principally in terms of framing biotic patterns over the E–C transition as an artifact of evolutionary patterns and stem- vs., crown-group diversity dynamics (i.e., suggesting that Ediacaran extinction and Cambrian explosion are different facets of the same process) – see discussion in the above section: ‘Do these intervals of biotic turnover represent extinctions at all?’.

## Key questions in 2023

Arguably, one of the most important questions surrounding Ediacaran extinction events is centered on establishing the magnitude of taxonomic loss over the E–C transition (i.e., to what extent these bioevents represent a ‘mass’ extinction) – something that will require better biostratigraphic correlations between fossil localities, and more sophisticated methods for extinction rate modeling. However, we argue that there are a number of other guiding questions which will help build more broad understanding for how pulses of extinction may be part of the sustained interval of biotic innovation that helped sculpt the more modern-looking Phanerozoic biosphere. These are summarized below:

### Were extinction pulses slow or rapid?

A crucial question that may eventually help distinguish between ‘catastrophe’ and ‘biotic replacement’ is whether late Ediacaran turnover pulses were rapid, or more protracted. Recent work on the Phanerozoic ‘Big 5’ mass extinctions has shown that the majority of these events were geologically rapid, with community collapse in those events unequivocally driven by environmental perturbation (i.e., the Permian–Triassic and Triassic-Jurassic) occurring on the order of 10^5^ years (Burgess et al., 2014; Erwin, 2014). In a ‘catastrophe’ scenario, therefore, we might expect late Ediacaran turnover pulses to be similarly fast. ‘Biotic replacement’, in contrast, would intuitively be a slower process, arguably operating over a range of evolutionary and ecological timescales as new organisms/behaviors evolve, become successful, disperse, and finally reach widespread ecological significance. Crucially, this model then predicts lengthy stratigraphic overlap between metazoans and Ediacara biota, with diversity decline beginning either once a key behavior emerges, or a threshold in ecosystem engineering intensity is reached. This point, for instance, stands in stark contrast to claims by Wood et al. (2019) that stratigraphic overlap comprises evidence against the ‘biotic replacement’ model. In other words, the lengthy co-existence of Ediacara biota and new ecosystem engineering behaviors is something *predicted* by ‘biotic replacement’, rather than a criterion for rejecting it. One immediate difficulty with answering this question is the paucity of fossiliferous sections that both span assemblage boundaries and possess sufficient age control. However, recent work on the Dengying Formation in South China has uncovered dickinsoniomorph fossils from near the base of the Shibantan Member (551–543 Ma) below the first occurrence of *Cloudina* (Xiao et al., 2020; Wang et al., 2021). The Shibantan Member thus likely spans the transition between the White Sea and Nama assemblages (Wang et al., 2021), and may offer an opportunity to constrain the timing of a putative first extinction pulse. Recent work from the Nagoryany Formation in Moldova (Francovschi et al., 2021) suggests that these strata preserve a similar interval, and so may offer another opportunity to study this transition in more detail.

### What were patterns of extinction selectivity and survivorship over the two turnover pulses?

Extinction events are characterized by ‘victims’ and ‘survivors’, the specific identities of which can offer vital clues as to the source(s) of ecological stress and thus help identify the proximal drivers of extinction. To provide a classic example, Knoll et al. (1996) showed over the Permian–Triassic mass extinction that heavily calcified invertebrates with low metabolic intensities suffered considerably higher extinction intensities than other groups; this pattern suggested that hypercapnia (linked to elevated CO_2_) was a likely culprit – an inference that pre-empted subsequent work establishing LIP volcanism and ocean acidification as overarching extinction drivers. Thus far, relatively little work has gone into analyzing patterns of selectivity over pulses of E–C extinction, beyond several authors noting that sessile, frondose, and semi-infaunal groups of Ediacara biota overwhelmingly survived the first pulse of extinction at the expense of groups that were mobile and/or surficial. Darroch et al. (2021) noted that sources of ecological stress associated with the specific impacts of bioturbation were hard to ally with the observed extinction selectivity patterns, although there remain big knowledge gaps surrounding what the ecosystem engineering impacts of early metazoans actually were (see question 4 below). In one of the few studies to directly address this question of selectivity, Evans et al. (2022) have noted that the overwhelming survivors of the first extinction pulse – the rangeomorphs and erniettomorphs – are characterized by high surface-area to volume (‘SA:V’) ratios (Laflamme et al., 2009). With the assumption that high SA:V ratios represent an adaptation (or advantage) to surviving in low-oxygen conditions, then the observed pattern of survivorship would be consistent with extinction driven by fluctuations in global redox conditions (see also Evans et al., 2018). We note that there are ambiguities surrounding to what extent the erniettomorphs had high surface areas exposed to the water column in life – most seem to have lived at least partially buried in the sediment (Ivantsov et al., 2016; Gibson et al., 2019; Darroch et al., 2022) – and whether their body plans were evolved for gas exchange as opposed to feeding (see Laflamme et al., 2013). Despite these caveats, Evans et al. (2022) demonstrate that focusing on selectivity is a powerful means for hypothesis testing, and a more complete knowledge of the physiological, paleoenvironmental, and paleoecological characteristics of ‘victims’ and ‘survivors’ over this boundary could offer vital hints as to what may have driven the first extinction pulse.

The second extinction pulse is similarly enigmatic. Although the vast majority of Ediacara biota disappear at, or shortly beneath, the base of the Cambrian worldwide, there are tantalizing hints that some taxa may persist for short intervals into the lower Cambrian (although we note the difficulties with defining the base of the Cambrian in some of these key sections – e.g., Nelson et al., 2022). However, there have been no convincing Ediacara biota reported from younger sediments, suggesting that survivors may be ‘dead clades walking’ – a characteristic of several Phanerozoic extinctions whereby some species persist in low numbers through an extinction only to die out early in the recovery interval (Jablonski, 2002; Hull et al., 2015). Among putative survivors, three examples stand out as being particularly convincing and/or in need of closer analysis: Jensen et al. (1998) figure apparent erniettomorph Ediacara biota from the Uratanna Formation in South Australia, stratigraphically above the FAD of *T. pedum.* In the southwestern United States, Hagadorn and Waggoner (2000) report forms similar to the erniettomorph taxon *Swartpuntia* from two separate localities above the FAD of *T. pedum*, and from strata that also preserve trilobites and archaeocyaths. Most recently, Nelson et al. (2022) report an erniettomorph from the Nomtsas Formation in the Neint Nababeep Plateau in South Africa. Although they interpreted this occurrence as Ediacaran, given that the Nomtsas Formation is traditionally interpreted as Cambrian (e.g., Linnemann et al., 2019), this may represent yet another survivor. Although these data are sparse (and have yet to be fully investigated beyond an initial description), a preliminary analysis of selectivity across this second extinction pulse may suggest that frondose erniettomorphs were survivors, while rangeomorph Ediacara biota were permanent casualties (although see Hoyal Cuthill, 2022 for an alternative view of rangeomorph extinction). If it is shown that erniettomorphs had significantly different paleobiologies or -ecologies than these other groups, then this may help establish extinction drivers over the E–C boundary itself.

### Are Ediacaran–Cambrian carbon isotope excursions recording global environmental perturbations?

Carbon isotope excursions in the late Ediacaran and earliest Cambrian are, largely, interpreted as recording perturbations to the global dissolved inorganic carbon (DIC) reservoir. As such, δ^13^C chemostratigraphy from carbonate successions has been used to correlate between sites regionally and globally, to construct age models, and to calibrate biostratigraphic changes across the E–C transition, particularly in the absence of radioisotopically constrained sections (e.g., Maloof et al., 2010; Bowyer et al., 2022). Although this approach has been applied widely to E–C studies, a number of geochemical studies of modern and ancient carbonate platforms have demonstrated the pitfalls of indiscriminate use of carbon isotope chemostratigraphy. Studies of modern carbonate platforms have shown that mineralogy and early marine diagenesis in shallow marine environments can result in variable δ^13^C records that are recording sediment- and fluid-buffered diagenetic precipitates that can be decoupled from global DIC (Swart, 2008; Oehlert and Swart, 2014; Higgins et al., 2018). Building upon these modern studies, chemostratigraphic data from Neoproterozoic carbonate platforms have demonstrated lateral δ^13^C variability across shelf to slope transects and, using the interpretive framework established in studies of modern carbonate platforms, have interpreted some of this variability as the result of early diagenesis (Ahm et al., 2019; Hoffman and Lamothe, 2019). Other studies of partially dolomitized Neoproterozoic platforms have demonstrated that dolomitizing fluids have the potential to alter δ^13^C values by up to 10‰ (Bold et al., 2020; Nelson et al., 2021). Finally, some detailed stratigraphic investigations have demonstrated a facies dependence on the character and/or preservation of carbon isotope excursions (e.g., Lu et al., 2013; Busch et al., 2022). Collectively, these studies highlight the need for careful assessment of the diagenetic histories of individual E–C carbonate platforms before records from individual sites can be interpreted within a global framework. With the recognition of more E–C carbon isotope excursions, some of which are only convincingly documented in a single region (see Figure 1), this regional scale assessment of diagenetic history is particularly important.

A parallel challenge for the community will be linking individual negative carbon isotope excursions that *are* established as global, to viable environmental perturbations that could result in an influx of light carbon. This challenge is not a new one. The extreme fluctuations in δ^13^C that characterize much of the Neoproterozoic have long been difficult to interpret in a mass-balance framework because of the dramatic changes in oxidants that are implied. Despite these long-standing challenges, rifting and rift-related volcanism of broadly E–C age occurred around the margins of Laurentia (Figure 3) and, as with some of the volcanic episodes associated with Phanerozoic mass extinctions, have recently been proposed as a possible “trigger” for a cascade of E–C environmental, ecological, and biotic effects (Hodgin et al., 2021; Smith et al., 2022). Similar to the historical trajectory of the study of Phanerozoic mass extinctions (e.g., Newell, 1967), the first step in testing the idea that E–C rift-related volcanism caused a perturbation(s) to geochemical cycles and a biotic crisis is demonstrating temporal coincidence among them. After temporal coincidence is established, the focus can turn to studying the geochemical response and the expected biotic selection across the E–C transition.

### What were the ecosystem engineering impacts of early animals?

The question as to how Ediacara biota and metazoans were interacting (discussed above) has yet to be fully answered, however, there are arguably more fundamental questions surrounding what the ecosystem engineering impacts of early animals actually were, and whether they were capable of driving large scale environmental change. While Cribb et al. (2019) focused on trace fossils and quantified their effects as indices of ‘ecosystem engineering impact’ (the ‘EEIs’ of Herringshaw et al., 2017), these may overestimate downstream effects, and so their use has been criticized (Minter et al., 2017). Other workers have highlighted the importance of biomixing vs. bioirrigation. For clarification, biomixing has relatively little ecosystem engineering impact (especially at shallow depths), whereas bioirrigation is a more powerful driver of environmental change – leading to deepening redox gradients, altered distribution of redox-sensitive elements, and increased availability of organic matter (e.g., Kristensen et al., 2012; Tarhan et al., 2015, 2021; Darroch et al., 2021). In this regard, the presence of treptichnid burrows in the late Ediacaran (Cribb et al., 2019; Jensen et al., 2000; Darroch et al., 2021) is crucial; treptichnids record the first substantial bioirrigative behaviors to appear in the trace fossil record (likely between 542.65–539.63 Ma; see age model in Nelson et al., 2022) and rapidly increase in size, complexity, and intensity through the latest Ediacaran and into the Cambrian. However, the actual downstream effects of this style of burrowing have not been measured, and so the significance of Ediacaran treptichnid-like behaviors is unknown. What is needed is a combination of in vivo ichnological experiments (encompassing a wide variety of different tracemakers) with integrated geochemical models; with these two approaches we might reasonably hope to understand what the significance of these behavioral innovations may have been (see, for example, Cribb et al., 2023).

Lastly, in addition to the record of bioturbation, there is a wealth of other ecosystem engineering impacts that appear at approximately the same time and should be more broadly considered (e.g., Erwin and Tweedt, 2012). Recent studies using fluid dynamics modeling have noted a paleoecological shift in the prevalence and character of inferred suspension feeders from the White Sea into the Nama intervals (Gibson et al., 2019;Cracknell et al., 2021; Darroch et al., 2022), which could have altered resource flows, and the distribution of habitable ecospace. Alternatively, it is also becoming apparent that passive predation is a mode of life that perhaps emerged as early as the Avalon assemblage (Liu et al., 2014; Dunn et al., 2022), but then may have expanded dramatically in the Nama (Bengston and Zhao, 1992; Darroch et al., 2016; Schiffbauer et al., 2016; Leme et al., 2022; Turk et al., 2022). Although not strictly an ecosystem engineering impact, the rise of predation was likely a source of ecological antagonistic stress that could have gradually marginalized the Ediacara biota, particularly if any of these groups possessed a mobile larval or dispersal stage early in development (Darroch et al., 2016). A prediction of this might be that Ediacaran communities from the Avalon through Nama assemblages show a noticeable shift from more neutral to niche-dominated processes in ecosystem dynamics – a facet of community paleoecology that can be readily preserved in the spatial distributions of fossils on bedding planes (see Mitchell et al., 2019, 2022).

### Bonus question: What were the ecosystem engineering impacts of Ediacara biota?

Finally, much discussion surrounding the ‘biotic replacement’ model has focused on the impact of metazoans and the emerging Cambrian-style fauna as ecosystem engineers. However, this conceptual approach ignores to what extent the evolving Ediacara biota may have been engaged in forms of ecosystem engineering themselves, and thus may be reinforcing a false narrative surrounding the character and drivers of turnover. Although none of the Ediacara biota are thought to have disrupted the sediment–water interface to the extent that Cambrian-style metazoans did, many might have engaged in other forms of ecosystem engineering. For example, as mentioned above, several groups of Ediacara biota were likely suspension feeders (Rahman et al., 2015; Gibson et al., 2019) and thus may have helped fuel the Cambrian explosion through forging energetic links between pelagic and benthic realms (Cracknell et al., 2021; Darroch et al., 2022), as well as help oxygenate the water column (Erwin and Tweedt, 2012). In addition, other groups of sessile – predominantly frondose – Ediacara biota apparently formed dense ‘meadows’ in both shallow and deep-water settings (see, e.g., Clapham et al., 2003; Droser and Gehling, 2008) which would have baffled currents, altered resource flows, and created a diversity of benthic niches and hydrodynamic refugia that could have been exploited by other taxa (analogous to modern seagrass meadows – see, e.g., Gartner et al., 2013). Lastly, Budd and Jensen (2017) have suggested that, following death and decay, patches of sessile Ediacara biota may have served as rich sources of organic matter – similar to whale falls – that in turn would have provided a selective pressure towards motility and the development of deposit-feeding strategies. Although some of these models are more plausible than others, they collectively emphasize that little is currently known about the ecosystem engineering impacts of the Ediacara biota. More broadly, these ideas illustrate that a key facet of understanding pulses of late Ediacaran extinction – both in terms of testing between hypothesized drivers and analyzing extinction selectivity – is understanding what different groups of Ediacara biota were actually doing within their ecosystems.

## Summary

Pulses of extinction in the late Ediacaran remain among the most enigmatic events in the history of life – occurring at a crucial interval during the Neoproterozoic rise of animals – and thus potentially influencing trends in early animal evolution as well as the character of the emerging, more modern-functioning and animal-dominated marine biosphere. The extent to which these events represent ‘mass extinctions’ – that is, the rapid disappearance of >70% of marine genera, rather than less severe and more protracted turnover pulses, is a question that doubtless requires a more focused analysis. Inferring the drivers of these putative extinction pulses is also a question that is fraught with difficulties stemming from biostratigraphic correlation and the interpretation of enigmatic geochemical signals. Despite this, the last 5 years of research has produced a wealth of new geological, paleontological, geochemical, and geochronological data that are slowly bringing this interval into focus. These new data have answered several of the questions posed by Darroch et al. (2018a), but have left others unanswered. Moreover, this work has led to new questions which promise to not only help unravel this critical interval in Earth’s history, but also contribute to our knowledge of extinction events more generally. Addressing the questions listed above will: (1) help link sources of environmental change in the late Ediacaran with drivers of ecological stress; and (2) explain patterns of extinction and survivorship across extinction pulses. Taken together, this new information will allow for a coherent picture of late Ediacaran extinction and help determine the role it may have played in the Cambrian explosion.

**Figure 4.**
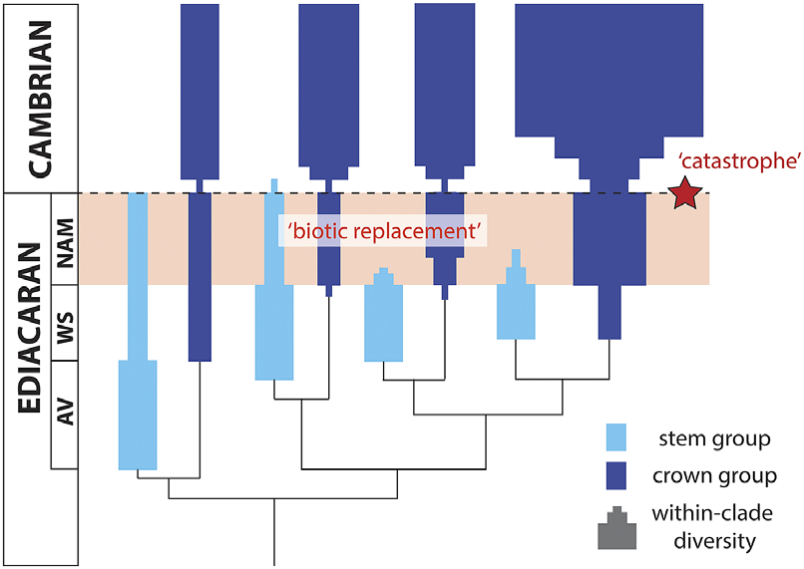
A hypothetical model for drivers of the E–C transition, modeled after Knoll and Carroll (1999); their figure 5). ‘AV’ = Avalon; ‘WS’ = White Sea; ‘NAM’ = Nama. This model interprets the majority of ‘Ediacara biota’ as stem-group metazoans, with a first pulse of extinction at the White Sea-Nama transition driven by the diversification – with associated downstream geobiological impacts – of crown-group metazoan clades (i.e., ‘biotic replacement’). A second pulse of extinction follows at the E–C boundary, driven by widespread environmental perturbation (i.e., ‘catastrophe’) following extensive rift volcanism. We note that this figure is strictly hypothetical, and the stem – and crown-groups depicted here do not intentionally correspond to specific biological groups shown in Figure 1.

Finally, the last 5 years of research allow us to propose a revised hypothesis for drivers of E–C biotic turnover, that can be tested and refined with further discoveries. Modeled after Knoll and Carroll (1999), the various tenets of this hypothesis build off a combination of growing consensus and current ideas, specifically: (1) the majority of ‘Ediacara biota’ are stem-group members of extant animal phyla, with crown group members of these same clades emerging as early as the Avalon assemblage (e.g., Dunn et al., 2018; Dunn et al., 2022); (2) in the absence of temporal correlation between isotope excursions and diversity loss (Rooney et al., 2020), a first pulse of extinction is recognized at the White Sea-Nama transition driven by competition, biotic interactions, and widespread geobiological change stemming from the diversification of crown-group metazoan clades (i.e., ‘biotic replacement’; see Darroch et al., 2015; Schiffbauer et al., 2016); and (3) a second pulse of extinction at the E–C boundary, potentially driven by widespread environmental perturbation (i.e., ‘catastrophe’) following extensive rift volcanism around the southern and southwestern margins of Laurentia (Hodgin et al., 2021; Smith et al., 2022) (Figure 4). This model thus hypothesizes a two-pulsed extinction of the Ediacara biota – with a first pulse driven by ‘biotic replacement’ and a second pulse driven by ‘catastrophe’ (i.e., environmental perturbation), combining recent evidence from paleontology, geochronology, and geochemistry. This model is obviously highly speculative and sensitive to the questions outlined above, but does frame testable hypotheses that can, and will, be targeted in the next decade of work. Testing this model will allow us to fold the biotic turnover events occurring over the E–C transition into broader discussions surrounding the tempo, mode, and drivers of mass extinction events, and invite comparisons with the ‘Big 5’ mass extinctions of the Phanerozoic.

## Supplementary material

For supplementary material accompanying this paper visit https://doi.org/10.1017/ext.2023.12. click here to view supplementary material

## References

[r1] Ahm A-SC, Maloof AC, Macdonald FA, Hoffman PF, Bjerrum CJ, Bold U, Rose CV, Strauss JV and Higgins JA (2019) An early diagenetic deglacial origin for basal Ediacaran “cap dolostones”. Earth and Planetary Science Letters 506, 292–307.

[r2] Algeo TJ and Scheckler SE (1998) Terrestrial-marine teleconnections in the Devonian: Links between the evolution of land plants, weathering processes, and marine anoxic events. Philosophical Transactions of the Royal Society of London. Series B: Biological Sciences 353, 113–130.

[r3] Alroy J (2008) Dynamics of origination and extinction in the marine fossil record. Proceedings of the National Academy of Sciences 105(supplement 1), 11536–11542. 10.1073/pnas.0802597105 PMC2556405 18695240

[r4] Alroy J (2014) Accurate and precise estimates of origination and extinction rates. Paleobiology 40, 374–397.

[r5] Amthor JE, Grotzinger JP, Shröder S, Bowring SA, Ramezani J, Martin MW and Matter A (2003) Extinction of *Cloudina* and *Namacalathus* at the Precambrian–Cambrian boundary in Oman. Geology 31, 431–434.

[r7] Bengston S and Zhao Y (1992) Predatorial borings in late Precambrian mineralized exoskeletons. Science 257, 367–369. 17832833 10.1126/science.257.5068.367

[r8] Boag TH, Darroch SAF and Laflamme M (2016) Ediacaran distributions in space and time: Testing assemblage concepts of earliest macroscopic body fossils. Paleobiology 42, 574–594.

[r9] Boddy CE, Mitchell EG, Merdith A and Liu AG (2022) Palaeolatitudinal distribution of the Ediacaran macrobiota. Journal of the Geological Society of London 179, jgs2021–030.

[r10] Bold U, Ahm A-SC, Schrag DP, Higgins JA, Jamsram E and Macdonald FA (2020) Effect of dolomitization on isotopic records from Neoproterozoic carbonates in southwestern Mongolia. Precambrian Research 350, 105902.

[r11] Bowring SA, Grotzinger JP, Condon DJ, Ramezani J, Newall MJ and Allen PA (2007) Geochronologic constraints on the chronostratigraphic framework of the Neoproterozoic Huqf Supergroup, Sultanate of Oman. American Journal of Science 307, 1097–1145.

[r12] Bowyer FT, Zhuravlev AY, Wood R, Shields GA, Zhou Y, Curtis A, Poulton SW, Condon DJ, Yang C and Zhu M (2022) Calibrating the temporal and spatial dynamics of the Ediacaran-Cambrian radiation of animals. Earth Science Reviews 225, 103913.

[r139] Brasier MD (1992) Background to the Cambrian explosion. Journal of the Geological Society 149, 585–587.

[r13] Brasier M, Cowie J and Taylor M (1994) Decision on the Precambrian-Cambrian boundary stratotype. Episodes Journal of International Geoscience 17, 3–8.

[r14] Buatois LA, Almond J, Mángano MG, Jensen S and Germs GJB (2018) Sediment disturbance by Ediacaran bulldozers and the roots of the Cambrian explosion. Scientific Reports 8, 4514. 29540817 10.1038/s41598-018-22859-9 PMC5852133

[r15] Buatois LA, Narbonne GM, Mángano MG, Carmona NB and Myrow P (2014) Relict ecosystems at the dawn of the Phanerozoic revolution. Nature Communications 5, 3544–3549. 10.1038/ncomms4544 24675373

[r16] Budd GE and Jensen S (2017) The origin of the animals and a ‘savannah’ hypothesis for early bilaterian evolution. Biological Reviews 92, 446–473. 26588818 10.1111/brv.12239

[r17] Budd GE and Mann RP (2020) The dynamics of stem and crown groups. Science Advances 6, eaaz1626. 32128421 10.1126/sciadv.aaz1626 PMC7030935

[r18] Burgess SD, Bowring S and Shen S (2014) High-precision timeline for Earth’s most severe extinction. Proceedings of the National Academy of Sciences 111, 3316–3321. 10.1073/pnas.1317692111 PMC3948271 24516148

[r19] Busch JF, Hodgin EB, Ahm A-SC, Husson JM, Macdonald FA, Bergmann KD, Higgins JA and Strauss JV (2022) Global and local drivers of the Ediacaran Shuram carbon isotope excursion. Earth and Planetary Science Letters 579, 117368.

[r20] Cai Y, Xiao S, Li G and Hua H (2019) Diverse biomineralizing animals in the terminal Ediacaran period herald the Cambrian explosion. Geology 47, 380–384.

[r149] Cañadas F, Papineau D, Leng ML and Li C (2022) Extensive primary production promoted the recovery of the Ediacaran Shuram excursion. Nature Communications 13, 148. 10.1038/s41467-021-27812-5 PMC8748710 35013337

[r146] Canfield De, Knoll AH, Poulton SW, Narbonne GM and Dunning GAR (2020) Carbon isotopes in clastic rocks and the Neoproterozoic carbon cycle. American Journal of Science 320, 97–124.

[r21] Chen Z, Zhou C, Yuan X and Xiao S (2019) Death march of a segmented and trilobate bilaterian elucidates early animal evolution. Nature 573, 412–415. 31485079 10.1038/s41586-019-1522-7

[r22] Clapham ME, Narbonne GM and Gehling JG (2003) Paleoecology of the oldest-known animal communities: Ediacaran assemblages at mistaken point, Newfoundland. Paleobiology 29, 527–544.

[r23] Clarkson MO, Kasemann SA, Wood RA, Lenton TM, Daines SJ, Richoz S, Ohnemueller F, Meixner A, Poulton SW and Tipper ET (2015) Ocean acidification and the Permo-Triassic mass extinction. Science 348, 229–232. 25859043 10.1126/science.aaa0193

[r24] Cracknell K, Ankor MJ, García-Bellido DC, Gehling JG, Darroch SAF and Rahman IA (2021) Pentaradial eukaryote suggests expansion of suspension feeding in White Sea-aged Ediacaran communities. Scientific Reports 11, 4121. 33602958 10.1038/s41598-021-83452-1 PMC7893023

[r25] Cribb AT, Kenchington CG, Koester B, Gibson BM, Boag TH, Racicot RA, Mocke H, Laflamme M and Darroch SAF (2019) Increase in metazoan ecosystem engineering prior to the Ediacaran-Cambrian boundary in the Nama group, Namibia. Royal Society Open Science 6, 190548. 31598294 10.1098/rsos.190548 PMC6774933

[r26] Cribb AT, van de Velde J, Berelson WM, Bottjer DJ and Corsetti FA (2023) Ediacaran-Cambrian bioturbation did not extensively oxygenate sediments in shallow-marine ecosystems. Geobiology 1–19. https://onlinelibrary.wiley.com/toc/14724669/0/0 10.1111/gbi.12550 36815223

[r27] Darroch SAF, Boag T, Racicot RA, Tweedt S, Mason SJ, Erwin DH and Laflamme M (2016) A mixed Ediacaran-metazoan assemblage from the Zaris sub-basin, Namibia. Palaeogeography, Palaeoclimatology, Palaeoecology 459, 198–208.

[r28] Darroch SAF, Cribb AT, Buatois LA, Germs GJB, Kenchington CG, Smith EF, Mocke H, O’Neil GR, Schiffbauer JD, Maloney KM, Racicot RA, Turk KA, Gibson BM, Almond J, Koester B, Boag TM, Tweedt SM and Laflamme M (2021) The trace fossil record of the Nama group, Namibia: Exploring the terminal Ediacaran roots of the Cambrian explosion. Earth Science Reviews 212, 103435.

[r29] Darroch SAF, Gibson BM, Syversen M, Rahman IA, Racicot RA, Dunn FS, Gutarra-Diaz S, Schindler E, Wehrmann A and Laflamme M (2022) The life and times of *Pteridinium simplex*. Paleobiology 48, 527–556.

[r30] Darroch SAF, Laflamme M and Wagner PJ (2018b) High ecological complexity in benthic Ediacaran ecosystems. Nature Ecology and Evolution 2, 1541–1547. 30224815 10.1038/s41559-018-0663-7

[r31] Darroch SAF, Smith EF, Laflamme M and Erwin DH (2018a) Ediacaran extinction and Cambrian explosion. Trends in Ecology and Evolution 33, 653–663. 30007844 10.1016/j.tree.2018.06.003

[r32] Darroch SAF, Sperling EA, Boag T, Racicot RA, Mason SJ, Morgan AS, Tweedt S, Myrow P, Erwin DH and Laflamme M (2015) Biotic replacement and mass extinction of the Ediacara biota. Proceedings of the Royal Society B 282, 20151003. 26336166 10.1098/rspb.2015.1003 PMC4571692

[r33] Droser ML and Gehling JG (2008) Synchronous aggregate growth in an abundant new Ediacaran tubular organism. Science 319, 1660–1662. 18356525 10.1126/science.1152595

[r34] Droser ML and Gehling JG (2015) The advent of animals: The view from the Ediacaran. Proceedings of the National Academy of Sciences 112, 4865–4870. 10.1073/pnas.1403669112 PMC4413262 25901306

[r137] Droser ML, Tarhan LG and Gehling JG (2017) The rise of animals in a changing environment: global ecological innovation in the late Ediacaran. Annual Review of Earth and Planetary Sciences 45, 593–617.

[r35] Dunn FS, Kenchington CG, Parry LA, Clark JW, Kendall RS and Wilby PR (2022) A crown-group cnidarian from the Ediacaran of Charnwood Forest, UK. Nature Ecology and Evolution 6, 1095–1104. 35879540 10.1038/s41559-022-01807-x PMC9349040

[r36] Dunn FS, Liu AG, Grazhdankin DV, Vixseboxse P, Flanerry-Sutherland J, Green E, Harris S, Wilby PR and Donoghue PCJ (2021) The developmental biology of *Charnia* and the eumetazoan affinity of the Ediacaran rangeomorphs. Science Advances 7, eabe0291. 34301594 10.1126/sciadv.abe0291 PMC8302126

[r37] Eden R, Manica A and Mitchell EG (2022) Metacommunity analyses show an increase in ecological specialisation throughout the Ediacaran period. PLoS Biology 20, e3001289. 35580078 10.1371/journal.pbio.3001289 PMC9113585

[r38] Erwin DH (2008) Macroevolution of ecosystem engineering, niche construction and diversity. Trends in Ecology and Evolution 23, 304–310. 18457902 10.1016/j.tree.2008.01.013

[r39] Erwin DH (2014) Temporal acuity and the rate and dynamics of mass extinctions. Proceedings of the National Academy of Sciences 111, 3203–3204. 10.1073/pnas.1400431111 PMC3948250 24522110

[r40] Erwin DH, Laflamme M, Tweedt SM, Sperling EA, Pisani D and Peterson KJ (2011) The Cambrian conundrum: Early divergence and later ecological success in the early history of animals. Science 334, 1091–1097. 22116879 10.1126/science.1206375

[r41] Erwin DH and Tweedt SM (2012) Ecological drivers of the Ediacaran-Cambrian diversification of Metazoa. Evolutionary Ecology 26, 417–433.

[r42] Evans SD, Diamond CW, Droser ML and Lyons TW (2018) Dynamic oxygen and coupled biological and ecological innovation during the second wave of the Ediacara biota. Emerging Topics in Life Sciences 2, 223–233. 32412611 10.1042/ETLS20170148

[r43] Evans SD, Tu C, Rizzo A, Suprenant RL, Boan PC, McCandless H, Marshall N, Xiao S and Droser ML (2022) Environmental drivers of the first major animal extinction across the Ediacaran White Sea-Nama transition. Proceedings of the National Academy of Sciences 119, e2207475119. 10.1073/pnas.2207475119 PMC9674242 36343248

[r143] Fike DA, Grotzinger JP, Pratt LM and Summons RE (2006) Oxidation of the Ediacaran ocean. Nature 444, 744–747. 17151665 10.1038/nature05345

[r44] Foote M (1999) Morphological diversity in the evolutionary radiation of Paleozoic and post-Paleozoic crinoids. Paleobiology 25, 1–115.

[r45] Francovschi I, Gradinaru E, Li H, Shumlyanskyy L and Ciobotaru V (2021) U–Pb geochronology and Hf isotope systematics of detrital zircon from the late Ediacaran Kalyus beds (east European platform): Palaeogeographic evolution of southwestern Baltica and constraints on the Ediacaran biota. Precambrian Research 355, 106062.

[r46] Gartner A, Tuya F, Lavery PS and McMahon K (2013) Habitat preferences of macroinvertebrate fauna among seagrasses with varying structural forms. Journal of Experimental Marine Biology and Ecology 439, 143–151.

[r47] Gehling JG (1991) The case for Ediacaran fossil roots to the metazoan tree. Geological Society of India Memoir 20, 181–224.

[r48] Gehling JG (1999) Microbial mats in terminal Proterozoic siliciclastics: Ediacaran death masks. PALAIOS 14, 40–57.

[r49] Germs GJB (1972) New shelly fossils from the Nama group, south West Africa. American Journal of Science 272, 752–761.

[r50] Gibson BM, Rahman I, Maloney K, Racicot R, Mocke H, Laflamme M and Darroch SAF (2019) Gregarious suspension feeding in a modular Ediacaran organism. Science Advances 5, eaaw0260. 31223655 10.1126/sciadv.aaw0260 PMC6584682

[r51] Gibson BM, Schiffbauer JD, Wallace AF and Darroch SAF (2023) The role of iron in the formation of Ediacaran ‘death masks’. Geobiology 1–14. 10.1111/gbi.12551 36843397

[r52] Glaessner MF (1984) The Dawn of Animal Life: A Biohistorical Study. New York: Cambridge University Press.

[r53] Gold DA, Runnegar B, Gehling JG and Jacobs DK (2015) Ancestral state reconstruction of ontogeny supports a bilaterian affinity for *Dickinsonia*. Evolution and Development 17, 315–324. 26492825 10.1111/ede.12168

[r54] Grazhdankin D (2004) Patterns of distribution in the Ediacaran biotas: Facies versus biogeography and evolution. Paleobiology 30, 203–221.

[r154] Grazhdankin DV (2014) Patterns of evolution of the Ediacaran soft-bodied biota. Journal of Paleontology 88, 269–283.

[r55] Grotzinger JP, Fike DA and Fischer WW (2011) Enigmatic origin of the largest-known carbon isotope excursion in Earth’s history. Nature Geoscience 4, 285–292.

[r56] Hagadorn JW and Waggoner B (2000) Ediacaran fossils from the southwestern Great Basin, United States. Journal of Paleontology 74, 349–359.

[r57] Herringshaw LG, Callow RT and McIlroy D (2017) Engineering the Cambrian explosion: The earliest bioturbators as ecosystem engineers. Geological Society of London Special Publications 448, 369–382.

[r58] Higgins JA, Blattler CL, Lundstrom EA, Santiago-Ramos D, Akhtar A, Ahm A-SC, Bialik O, Holmden C, Bradbury H, Murray ST and Swart P (2018) Mineralogy, early marine diagenesis, and the chemistry of shallow water carbonate sediments. Geochimica et Cosmochimica Acta 220, 512–534.

[r59] Hodgin EB, Nelson LL, Wall CJ, Barron-Diaz AJ, Webb LC, Schmitz MD, Fike DA, Hagadorn JW and Smith EF (2021) A link between rift-related volcanism and end-Ediacaran extinction? Integrated chemostratigraphy, biostratigraphy, and U-Pb geochronology from Sonora, Mexico. Geology 49, 115–119.

[r60] Hoffman PF and Lamothe KG (2019) Seawater-buffered diagenesis, destruction of carbon isotope excursions, and the composition of DIC in Neoproterozoic oceans. Proceedings of the National Academy of Sciences USA 116, 18874–18879. 10.1073/pnas.1909570116 PMC6754552 31484778

[r61] Hoyal Cuthill J (2022) Ediacaran survivors in the Cambrian: suspicions, denials and a smoking gun. Geological Magazine 159, 1210–1219.

[r62] Hull PM, Darroch SAF and Erwin DH (2015) Rarity in mass extinctions and the future of ecosystems. Nature 528, 345–351. 26672552 10.1038/nature16160

[r63] Husson JM, Higgins JA, Maloof AC and Schoene B (2015) Ca and mg isotope constraints on the origin of Earth’s deepest δ13C excursion. Geochimica et Cosmochimica Acta 160, 243–266.

[r142] Ireland TR, Flöttmann T, Fanning CM, Gibson GM and Preiss WV (1998) Development of the early Paleozoic Pacific margin of Gondwanafrom detrital-zircon ages across the Delamerian orogen. Geology 26, 243–246.

[r152] Ivantsov AY, Narbonne GM, Trusler PW, Greentree C and Vickers-Rich P (2016) Elucidating Ernietta: new insights from exceptional specimens in the Ediacaran of Namibia. Lethaia 49, 540–554.

[r64] Jablonski D (2002) Survival without recovery after mass extinctions. Proceedings of the National Academy of Sciences USA 99, 8139–8144. 10.1073/pnas.102163299 PMC123034 12060760

[r65] Jensen S, Gehling JG and Droser ML (1998) Ediacara-type fossils in Cambrian sediments. Nature 393, 567–569.

[r66] Jensen S, Saylor BZ, Gehling JG and Germs GJB (2000) Complex trace fossils from the terminal Proterozoic of Namibia. Geology 28, 143–146.

[r67] Jones CG, Lawton JH and Shachak M (1994) Organisms as ecosystem engineers. Oikos 69, 373–386.

[r68] Kirschvink JL, Magaritz M, Ripperdan RL, Zhuravlev AY and Rozanov AY (1991) The Precambrian-Cambrian boundary: Magnetostratigraphy and carbon isotopes resolve correlation problems between Siberia, Morocco, and South China. GSA Today 1, 69–91.

[r69] Knauth LP and Kennedy MJ (2009) The late Precambrian greening of the earth. Nature 460, 728–732. 19587681 10.1038/nature08213

[r70] Knoll AH, Bambach RK, Canfield DE and Grotzinger JP (1996) Comparative earth history and late Permian mass extinction. Science 273, 452–457. 11541222

[r71] Knoll AH and Carroll SB (1999) Early animal evolution: Emerging views from comparative biology and geology. Science 284, 2129–2137. 10381872 10.1126/science.284.5423.2129

[r72] Knoll AH and Walter MR (1992) Latest Proterozoic stratigraphy and earth history. Nature 356, 673–678. 11537861 10.1038/356673a0

[r73] Kocsis ÁT, Reddin CJ, Alroy J and Kiessling W (2019) The R package divDyn for quantifying diversity dynamics using fossil sampling data. Methods in Ecology and Evolution 10, 735–743.

[r74] Kristensen E, Penha-Lopes G, Delefosse M, Valdemarsen T, Quintana CO and Banta GT (2012) What is bioturbation? The need for a precise definition for fauna in aquatic sciences. Marine Ecology Progress Series 446, 285–302.

[r75] Krug AZ and Jablonski D (2012) Long-term origination rates are reset only at mass extinctions. Geology 40, 731–734.

[r145] Ksepka DT, Stidham TA and Williamson TE (2017) Early Paleocene landbird supports rapid phylogenetic and morphological diversification of crown birds after the K-Pg mass extinction. Proceedings of the National Academy of Sciences 114, 8047–8052. 10.1073/pnas.1700188114 PMC5544281 28696285

[r76] Laflamme M, Darroch SAF, Tweedt SM, Peterson KJ and Erwin DH (2013) The end of the Ediacara biota: Extinction, biotic replacement, or Cheshire cat? Gondwana Research 23, 558–573.

[r158] Laflamme M, Xiao S and Kowalewski M (2009) Osmotrophy in modular Ediacara organisms. Proceedings of the national Academy of Sciences 106, 14438–14443. 10.1073/pnas.0904836106 PMC2732876 19706530

[r77] Leme JM, Van Iten H and Simoes MG (2022) A new conulariid (Cnidaria, Scyphozoa) from the terminal Ediacaran of Brazil. Frontiers in Earth Science 10, 777746. 10.3389/feart.2022.777746.

[r78] Li Z, Cao M, Lloyd SJ, Algeo TJ, Zhao H, Wang X, Zhao L and Chen Z-Q (2020) Transient and stepwise ocean oxygenation during the late Ediacaran Shuram excursion: Insights from carbonate δ^238^U of northwestern Mexico. Precambrian Research 344, 105741.

[r79] Linnemann U, Ovtcharova M, Schaltegger U, Gartner A, Hautmann M, Geyer G, Vickers-Rich P, Rich T, Plessen B, Hofmann M, Zieger J, Krause R, Kriesfield L and Smith J (2019) New high-resolution age data from the Ediacaran–Cambrian boundary indicate rapid, ecologically driven onset of the Cambrian explosion. Terra Nova 31, 49–58.

[r80] Liu AG, Kenchington CG and Mitchell EG (2015) Remarkable insights into the paleoecology of the Avalonian Ediacaran macrobiota. Gondwana Research 27, 1355–1380.

[r81] Liu AG, Matthews JJ, Menon LR, McIlroy D and Brasier MD (2014) *Haootia quadriformis* n. gen., n. sp., interpreted as a muscular cnidarian impression from the Late Ediacaran period (approx. 560 Ma). Proceedings of the Royal Society B 281, 20141202. 25165764 10.1098/rspb.2014.1202 PMC4173675

[r82] Love GD and Zumberge JA (2021) Emerging patterns in Proterozoic lipid biomarkers: Implications for marine biospheric evolution and the ecological rise of eukaryotes. In Lyons T, Turchyn A and and Reinhard C (eds.), Elements in Geochemical Tracers in Earth System Science. Cambridge: Cambridge University Press.

[r83] Lu M, Lu Y, Ikejiri T, Sun D, Carroll R, Blair EH, Algeo TJ and Sun Y (2021) Periodic oceanic euxinia and terrestrial fluxes linked to astronomical forcing during the late Devonian Frasnian–Famennian mass extinction. Earth and Planetary Science Letters 562, 116839.

[r160] Lu M, Zhu M, Zhang J, Shields-Zhou G, Li G, Zhao F, Zhao X and Zhao M (2013) The DOUNCE event at the top of the Ediacaran Doushantuo Formation, South China: Broad stratigraphic occurrence and non-diagenetic origin. Precambrian Research 225, 86–109.

[r84] Macdonald FA, Strauss JV, Sperling EA, Halverson GP, Narbonne GM, Johnston DT, Kunzmann M, Schrag DP and Higgins JA (2013) The stratigraphic relationship between the Shuram carbon isotope excursion, the oxygenation of Neoproterozoic oceans, and the first appearance of the Ediacara biota and bilaterian trace fossils in northwestern Canada. Chemical Geology 362, 250–272.

[r85] Maloof AC, Porter SM, Moore JL, Dudas FO, Bowring SA, Higgins JA, Fike DA and Eddy MP (2010) The earliest Cambrian record of animals and ocean geochemical change. GSA Bulletin 122, 1731–1774.

[r86] Mángano MG and Buatois LA (2014) Decoupling of body-plan diversification and ecological structuring during the Ediacaran-Cambrian transition: Evolutionary and geobiological feedbacks. Proceedings of the Royal Society B 281, 20140038. 24523279 10.1098/rspb.2014.0038 PMC4027402

[r87] Mángano MG and Buatois LA (2020) The rise and early evolution of animals: Where do we stand from a trace-fossil perspective? Interface Focus 10, 20190103. 32642049 10.1098/rsfs.2019.0103 PMC7333897

[r88] Marenco KN and Bottjer DJ (2007) Ecosystem engineering in the fossil record: Early examples from the Cambrian Period. In Cuddington K, Byers JE, Wilson WG and Hastings A (eds.), Ecosystem Engineers: Plants to Protists. Amsterdam: Academic Press, pp. 163–183.

[r147] Matthews JJ, Liu AG, Yang C, McIlroy D, Levell B and Condon DJ (2021) A Chronostratigraphic framework for the rise of the Ediacaran Macrobiota: new constraints from Mistaken Point Ecological Reserve, Newfoundland. Geological Society of America Bulletin 133, 612–624.

[r89] Meysman FJR, Middelburg JJ and Heip CHR (2006) Bioturbation: A fresh look at Darwin’s last idea. Trends in Ecology and Evolution 21, 688–695. 16901581 10.1016/j.tree.2006.08.002

[r153] Minter NJ, Buatois LA, Mangano MG, Davies NS, Gibling MR, Macnaughton RB and Labandeira CC (2017) Early bursts of diversification defined the faunalcolonization of land. Nature Ecology and Evolution 1075.

[r90] Mitchell EG, Evans S, Chen Z and Xiao S (2022) A new approach for investigating spatial relationships of ichnofossils: A case study of Ediacaran–Cambrian animal traces. Paleobiology 48, 557–575.

[r91] Mitchell EG, Harris S, Kenchington CG, Vixseboxse P, Roberts L, Clark C, Dennis A, Liu AG and Wilby PR (2019) The importance of neutral over niche processes in structuring Ediacaran early animal communities. Ecology Letters 22, 2028–2038. 31515929 10.1111/ele.13383 PMC6899650

[r92] Muscente AD, Bykova N, Boag TH, Buatois LA, Mángano GM, Eleish A, Prabhu A, Pan F, Meyer MB, Schiffbauer JD, Fox P, Hazen RM and Knoll AH (2019) Ediacaran biozones identified with network analysis provide evidence for pulsed extinctions of early complex life. Nature Communications 10, 911. 10.1038/s41467-019-08837-3 PMC6384941 30796215

[r93] Narbonne GM (2005) The Ediacara biota: Neoproterozoic origin of animals and their ecosystems. Annual Reviews of Earth and Planetary Sciences 33, 421–442.

[r94] Narbonne GM, Kaufman AJ and Knoll AH (1994) Integrated chemostratigraphy and biostratigraphy of the Windermere Supergroup, northwestern Canada: Implications for Neoproterozoic correlations and the early evolution of animals. Geological Society of America Bulletin 106, 1281–1292. 11539403 10.1130/0016-7606(1994)106<1281:icabot>2.3.co;2

[r159] Nelson LL, Ahm A-SC, Macdonald FA, Higgins JA and Smith EF (2021) Fingerprinting local controls on the Neoproterozoic carbon cycle with the isotopic record of Cryogenian carbonates in the Panamint Range, California. Earth and Planetary Science Letters 566, 116956.

[r95] Nelson LL, Ramezani J, Almond JE, Darroch SAF, Taylor WL, Brenner DC, Furey RP, Turner M and Smith EF (2022) Pushing the boundary: A calibrated Ediacaran–Cambrian stratigraphic record from the Nama group in northwestern Republic of South Africa. Earth and Planetary Science Letters 580, 117396.

[r96] Newell ND (1967) Revolutions in the history of life. Geological Society of America Special Publications 89, 63–91.

[r144] O’Leary MA, Bloch JI, Flynn JJ, Gaudin TJ, Giallombardo A, Giannini NP, Goldberg SL, Kraatz BP, Luo Z-X, Meng J, Ni X, Novacek MJ, Perini FA, Randall ZS, Rougier GW, Sargis EJ, Silcox MT, Simmons NB, Spaulding M, Velazco PM, Weksler M, Wible JR and Cirranello AL (2013) The Placental Mammal Ancestor and the Post K-Pg Radiation of Placentals. Science 339, 662–667. 23393258 10.1126/science.1229237

[r97] Oehlert AM and Swart PK (2014) Interpreting carbonate and organic carbon isotope covariance in the sedimentary record. Nature Communications 5, 4672. 10.1038/ncomms5672 25135457

[r98] Ouyang Q, Zhou C, Xiao S, Chen Z and Shao Y (2019) Acanthomorphic acritarchs from the Ediacaran Doushantuo formation at Zhangcunping in South China, with implications for the evolution of early Ediacaran eukaryotes. Precambrian Research 320, 171–192.

[r99] Park T-YS, Jung J, Lee M, Lee S, Zhen YY, Hua H, Warren LV and Hughes NC (2021) Enduring evolutionary embellishment of cloudinids in the Cambrian. Royal Society Open Science 8, 34909213. 10.1098/rsos.210829 PMC8652264 34909213

[r100] Parry LA, Boggiani PC, Condon DJ, Garwood RJ, Leme JD-M, McIlroy D, Brasier MD, Trindade R, Campanha GAC, Pacheco MLAF, Diniz CQC and Liu AG (2017) Ichnological evidence for meiofaunal bilaterians from the terminal Ediacaran and earliest Cambrian of Brazil. Nature Ecology and Evolution 10, 1455–1464. 10.1038/s41559-017-0301-9. 29185521

[r101] Pineda-Munoz S, Wang Y, Lyons SK, Toth AB and McGuire JL (2021) Mammal species occupy different climates following the expansion of human impacts. Proceedings of the National Academy of Sciences 118, e1922859118. 10.1073/pnas.1922859118 PMC7812786 33397717

[r102] Pu JP, Bowring SA, Ramezani J, Myrow P, Raub TD, Landing E, Mills A, Hodgin E and Macdonald FA (2016) Dodging snowballs: Geochronology of the Gaskiers glaciation and the first appearance of the Ediacaran biota. Geology 44, 955–958.

[r103] Rahman IA, Darroch SAF, Racicot RA and Laflamme M (2015) Suspension feeding in the enigmatic Ediacaran organism *Tribrachidium* demonstrates complexity of Neoproterozoic ecosystems. Science Advances 1, e1500800. 26702439 10.1126/sciadv.1500800 PMC4681345

[r104] Raup DM and Sepkoski JJ (1982) Mass extinctions in the marine fossil record. Science 215, 1501–1503. 17788674 10.1126/science.215.4539.1501

[r105] Rhoads DC, Yingst JY and Ullman WJ (1978) Seafloor stability in Central Long Island sound: Part I. temporal changes in erodibility of fine-grained sediment. In Wiley ML (ed.), Estuarine Interactions. New York: Academic Press, pp. 221–244.

[r106] Rooney AD, Cantine MD, Bergmann KD, Gomez-Perez I, Al Baloushi B, Boag TH, Busch JF, Sperling EA and Strauss JV (2020) Calibrating the coevolution of Ediacaran life and environment. Proceedings of the National Academy of Sciences USA 117, 16824–16830. 10.1073/pnas.2002918117 PMC7382294 32632000

[r107] Rosenberg R, Nilsson HC and Diaz RJ (2001) Response of benthic fauna and changing sediment redox profiles over a hypoxic gradient. Estuarine and Coastal Shelf Science 53, 343–350.

[r108] Ruhl M, Bonis NR, Reichert G-J, Damste JSS and Kurschner WM (2011) Atmospheric carbon injection linked to end-Triassic mass extinction. Science 333, 430–434. 21778394 10.1126/science.1204255

[r109] Schiffbauer JD, Huntley JW, O’Neil GR, Darroch SAF, Laflamme M and Cai Y (2016) The latest Ediacaran wormworld fauna: Setting the ecological stage for the Cambrian explosion. GSA Today 26, 4–11.

[r110] Schiffbauer JD, Rosbach S, Pulsipher MA, Leibach W, Nolan M, Tang Q, Lindsay-Kauffman A and Selly T (2022). Diversity in the age of tubes: A morphometric approach to understanding the breadth of Ediacaran tube-dwelling taxa. Geological Society of America Abstracts with Programs 54. doi: 10.1130/abs/2022AM-381858

[r140] Schiffbauer JD, Selly T, Jacquet SM, Merz RA, Nelson LL, Strange MA, Cai Y and Smith EF (2020) Discovery of bilaterian-type through-guts in cloudinomorphs from the terminal Ediacaran Period: Nature Communications 11, 1–12. 10.1038/s41467-019-13882-z PMC6954273 31924764

[r111] Seilacher A (1984) Late Precambrian and early Cambrian Metazoa: Preservational or real extinctions? In Holland HD and Trendall AF (eds.), Patterns of Change in Earth Evolution. Heidelberg, Germany: Springer-Verlag, pp. 159–168.

[r112] Seilacher A (1985) Discussion of Precambrian metazoans. Philosophical Transactions of the Royal Society of London Series B 311, 47–48.

[r113] Seilacher A (1989) Vendozoa: Organismic construction in the Proterozoic biosphere. Lethaia 22, 229–239.

[r114] Seilacher A (1992) Vendobionta and Psammocorallia: Lost constructions of Precambrian evolution. Journal of the Geological Society of London 149, 607–613.

[r115] Shen SZ, Crowley JL, Wang Y, Bowring SA, Erwin DH, Sadler PM, Cao C-Q, Rothman DH, Henderson CM, Ramezani J, Zhang H, Shen Y, Wang X-D, Wang W, Mu L, Li Y-G, Liu Z-L, Liu L-J, Zheng Y, Y-F J and Jin Y-G (2011) Calibrating the end-Permian mass extinction. Science 334, 1367–1372. 22096103 10.1126/science.1213454

[r116] Shore AJ, Wood RA, Butler IB, Zhuravelev AY, McMahon S, Curtis A and Bowyer FT (2021) Ediacaran metazoan reveals lophotrochozoan affinity and deepens root of Cambrian explosion. Science Advances 7, eabf2933. 10.1126/sciadv.abf293. 33523867 PMC7775780

[r117] Shore AJ, Wood RA, Curtis A and Bowyer FT (2020) Multiple branching and attachment structures in cloudinomorphs, Nama Group, Namibia. Geology 48, 877–881.

[r118] Slagter S, Hao W, Planavsky NJ, Konhauser KO and Tarhan LG (2022) Biofilms and agents of Ediacara-style fossilization. Scientific Reports 12, 8631. 35606399 10.1038/s41598-022-12473-1 PMC9127100

[r119] Smith EF, Nelson LL, O’Connell N, Eyster AE and Lonsdale MC (2023) The Ediacaran− Cambrian transition in the southern Great Basin, United States. GSA Bulletin 135, 1393–1414.

[r120] Smith EF, Nelson LL, Strange MA, Eyster AE, Rowland SM, Schrag DP and Macdonald FA (2016) The end of the Ediacaran: Two exceptionally preserved body fossil assemblages from Mount Dunfee, Nevada, USA. Geology 44, 911–914.

[r121] Swart P (2008) Global synchronous changes in the carbon isotopic composition of carbonate sediments unrelated to changes in the global carbon cycle. Proceedings of the National Academy of Sciences USA 105, 13741–13745. 10.1073/pnas.0802841105 PMC2544524 18772393

[r122] Tarhan LG, Droser ML, Cole DB and Gehling JG (2018) Ecological expansion and extinction in the late Ediacaran: Weighing the evidence for environmental and biotic drivers. Integrative and Comparative Biology 58, 688–702. 29718307 10.1093/icb/icy020

[r123] Tarhan LG, Droser ML, Planavsky NJ and Johnston DT (2015) Protracted development of bioturbation through the early Palaeozoic era. Nature Geoscience 8, 865–869.

[r124] Tarhan LG, Zhao M and Planavsky NJ (2021) Bioturbation feedbacks on the phosphorous cycle. Earth and Planetary Science Letters 556, 116961.

[r125] Topper T, Betts MJ, Dorjnamjaa D, Li G, Li L, Altanshagai G, Enkhbaatar B and Skovsted CB (2022) Locating the BACE of the Cambrian: Bayan Gol in southwestern Mongolia and global correlation of the Ediacaran–Cambrian boundary. Earth Science Reviews 226, 104017.

[r126] Turk KA, Maloney KM, Laflamme M and Darroch SAF (2022) Paleontology and ichnology of the late Ediacaran Nasep-Huns transition (Nama group, southern Namibia). Journal of Paleontology 96, 753–769.

[r150] Verdel C, Wernicke BP and Bowring SA (2011) The Shuram and subsequent Ediacaran carbon isotope excursions from southwest Laurentia, and implications for environmental stability during the metazoan radiation. Geological Society of America Bulletin 123, 1539–1559.

[r127] Waggoner B (1999) Biogeographic analyses of the Ediacara biota: A conflict with paleotectonic reconstructions. Paleobiology 25, 440–458.

[r128] Waggoner B (2003) The Ediacaran biotas in space and time. Integrative and Comparative Biology 43, 104–113. 21680415 10.1093/icb/43.1.104

[r129] Wang X, Chen Z, Pang K, Chuanming Z, Xiao S, Wan B and Yuan X (2021) *Dickinsonia* from the Ediacaran Dengying formation in the Yangtze gorges area, South China. Palaeoworld 30, 602–609.

[r138] Wood RA and Curtis A (2014) Extensive metazoan reefs from the Ediacaran Nama Group, Namibia: the rise of benthic suspension feeding. Geobiology 13, 112–122. 25556318 10.1111/gbi.12122

[r131] Wood RA, Liu AG, Bowyer F, Wilby PR, Dunn FS, Kenchington CG, Hoyal-Cuthill JF, Mitchell EG and Penny A (2019) Integrated records of environmental change and evolution challenge the Cambrian explosion. Nature Ecology and Evolution 3, 528–538. 30858589 10.1038/s41559-019-0821-6

[r132] Wood RA, Poulton SW, Prave AR, Hoffmann K-H, Clarkson MO, Guilbaud R, Lyne JW, Tostevin R, Bowyer F, Penny AM, Curtis A and Kasemann SA (2015) Dynamic redox conditions control late Ediacaran metazoan ecosystems in the Nama group, Namibia. Precambrian Research 261, 252–271.

[r157] Xiao S, Narbonne GM, Zhou C, Laflamme M, Grazhdankin D, Moczydlowska-Vidal M and Cui H (2016) Towards an Ediacaran time scale: problems, protocols, and prospects. Episodes 36, 540–555.

[r133] Xiao S, Chen Z, Pang K, Zhou C and Yuan X (2020) The Shibantan Lagerstätte: Insights into the Proterozoic–Phanerozoic transition. Journal of the Geological Society 178, jgs2020–135.

[r155] Yang B, Steiner M, Schiffbauer JD, Selly T, Wu X, Zhang C and Liu P (2020) Ultrastructure of Ediacaran cloudinids suggests diverse taphonomic histories and affinities with non-biomineralized annelids. Scientific Reports 10, 535. 31953458 10.1038/s41598-019-56317-x PMC6968996

[r156] Yang B, Steiner M, Zhu M, Guoxiang L, Liu J and Liu P (2016) Transitional Ediacaran–Cambrian small skeletal fossil assemblages from South China and Kazakhstan: implications for chronostratigraphy and metazoan evolution. Precambrian Research 285, 202–215.

[r134] Yang C, Rooney AD, Condon DJ, Li X-H, Grazhdankin DV, Bowyer FT, Hu C, Macdonald FA and Zhu M (2021) The tempo of Ediacaran evolution. Science Advances 7, eabi9643. 34731004 10.1126/sciadv.abi9643 PMC8565906

[r135] Yeakel JD, Pires MM, de Aguiar MAM, O’Donnell JL, Guimaraes PR, Gravel D and and Gross T (2020) Diverse interactions and ecosystem engineering can stabilize community assembly. Nature Communications 11, 3307. 10.1038/s41467-020-17164-x PMC7335095 32620766

[r136] Youbi N, Ernst RE, Söderlund U, Boumehdi MA, Lahna A, Tassinari CCG, El Moume W and Bensalah MK (2020) The Central Iapetus magmatic province (CIMP): An updated review and link with the c. 580 ma Gaskiers glaciation. In Adatte, T., Bond, D.P.G., and Keller, G., eds. New Developments on Volcanism, Impacts and Mass Extinctions. Geological Society of America Special Paper 544.

[r148] Zhang F, Xiao S, Romaniello SJ, Hardisty D, Li C, Melezhik V, Pokrovsky B, Cheng M, Shi W, Lenton TM and Anbar AD (2019) Global marine redox changes drove the rise and fall of the Ediacara biota. Geobiology 17, 594–610. 31353777 10.1111/gbi.12359 PMC6899691

[r141] Zhu M, Zhuravlev AY, Wood RA, Zhao F and Sukhov SS (2017) A deep root for the Cambrian explosion: implications of a new bio- and chemostratigraphy from the Siberian Platform. Geology 45, 459–462.

